# Systematic determination of disulfide bond reduction potentials reveals a nonequilibrium redox hierarchy in cyanobacteria

**DOI:** 10.1073/pnas.2600150123

**Published:** 2026-05-19

**Authors:** Kenya Tanaka, Akihiko Kondo, Tomohisa Hasunuma

**Affiliations:** ^a^Engineering Biology Research Center, Kobe University, Nada, Kobe 657-8501, Japan; ^b^Graduate School of Science, Technology and Innovation, Kobe University, Nada, Kobe 657-8501, Japan; ^c^Research Center for Solar Energy Chemistry, Graduate School of Engineering Science, Osaka University, Toyonaka, Osaka 560-8531, Japan; ^d^Biomanufacturing Design Research Team, RIKEN Center for Sustainable Resource Science, Yokohama, Kanagawa 230-0045, Japan; ^e^Department of Chemical Science and Engineering, Faculty of Engineering, Kobe University, Nada, Kobe 657-8501, Japan

**Keywords:** redox proteomics, disulfide bond, thioredoxin, Calvin-Benson-Bassham cycle, AlphaFold

## Abstract

Disulfide bonds act as reversible switches that regulate cellular function in nearly all organisms. Their behavior is set by the midpoint potential (*E*_m_), yet *E*_m_ is known for only a small number of sites, mostly in purified proteins studied away from their natural partners. We developed a mass-spectrometry workflow that measures *E*_m_ directly from native cell lysates. By equilibrating proteins of the cyanobacterium *Synechocystis* sp. PCC 6803 in defined redox buffers and reading out the oxidation state of individual cysteines, we obtained 368 *E*_m_ values across the proteome and validated them against purified proteins. A key example is the regulatory protein CP12: its *E*_m_ in isolation differs strongly from the value measured in lysate and converges only when its physiological partner, thioredoxin, is included, showing that our approach captures the effective potentials that operate inside cells. Combining *E*_m_ with absolute measurements of cysteine redox state in light and darkness, we mapped intracellular redox “operating points” for Calvin–Benson–Bassham (CBB) cycle enzymes. Phosphoribulokinase sits near thioredoxin, whereas fructose 1,6-bisphosphatase/sedoheptulose 1,7-bisphosphatase (F/SBPase) and CP12 are maintained at more oxidized, nonequilibrium states. These results reveal a hierarchical redox control network in photosynthetic metabolism and provide a general strategy for measuring context-dependent redox switches in living systems.

## Significance

Disulfide bonds play diverse roles in proteins, ranging from structural stabilization to reversible regulation of enzyme activity, and they are central to controlling the CBB cycle in photosynthetic organisms. How easily each bond changes state is set by its reduction potential (*E*_m_), yet *E*_m_ is known for only limited sites and usually measured in purified proteins removed from their natural partners. We developed a lysate-based redox proteomics method that determines hundreds of *E*_m_ values across the cyanobacterial proteome in a native mixture of proteins. These data reveal a nonequilibrium hierarchy around thioredoxin: phosphoribulokinase operates near thioredoxin, whereas other CBB cycle enzymes are kept more oxidized. Our approach provides a broadly applicable strategy to quantify context-dependent *E*_m_ that organize cellular metabolism.

Protein disulfide bonds form by oxidation of a pair of cysteine residues. The resulting side-chain crosslinks can stabilize protein structure and support proper folding, which can secondarily increase thermostability (1). Reversible disulfide formation and cleavage operate broadly as switches for protein function (2). In addition, cysteine pairs participate in antioxidant defense by reducing reactive oxygen species (3). Thus, the redox state of disulfide–cysteine pairs underpins diverse cellular roles, spanning thermal stabilization, catalytic control, and detoxification.

A central thermodynamic descriptor for a disulfide switch is its midpoint (reduction) potential (*E*_m_), which describes the equilibrium tendency of a given disulfide/dithiol couple to exist in the reduced or oxidized state. Reported *E*_m_ values for protein disulfides cover a wide range (approximately −470 to −89 mV), indicating substantial diversity in redox sensitivity (4). Disulfides that primarily stabilize a functional fold often tend to be relatively refractory to reduction and therefore show more negative *E*_m_ values, whereas regulatory disulfides that cycle on biologically relevant timescales are expected to have *E*_m_ closer to the prevailing intracellular environment. Consequently, *E*_m_ is a key parameter that helps determine function and positioning of each disulfide within redox networks while the physiological direction and efficiency of redox regulation also depend on kinetic coupling and network context (5).

In photosynthetic organisms—including plants, algae, and cyanobacteria—reversible cysteine oxidation controls numerous reactions and even transcription/translation steps (6–9). A canonical example is the Calvin–Benson–Bassham (CBB) cycle, whose activity is tuned by light. Upon illumination, the photosynthetic electron transport chain reduces ferredoxin, generating NADPH and supplying reducing power for CO_2_ fixation (10). A portion of this flux is channeled via ferredoxin–thioredoxin reductase (FTR) to thioredoxin (Trx), which in plants and eukaryotic algae reduces regulatory disulfides in fructose-1,6-bisphosphatase (FBPase), phosphoribulokinase (PRK), sedoheptulose-1,7-bisphosphatase (SBPase), and GAPDH, thereby activating the CBB cycle (6). In the dark, reducing input from the photosynthetic electron transport chain ceases and the balance shifts toward oxidative processes. As a result, oxidation of target cysteine pairs, including through H_2_O_2_-dependent pathways, can become dominant and inactivate CBB-cycle enzymes (11–13). In cyanobacteria, the same Fd–FTR–Trx logic applies (with TrxA as a major isoform), and a bifunctional fructose-1,6-biphosphatase/sedoheptulose-1,7-biphosphatase (F/SBPase), CP12, and PRK carry reversible disulfides implicated in CBB control (7); however, their energetic placement relative to TrxA and oxidative partners in the native network remains insufficiently quantified, especially under changing light conditions. This light-dependent control balances NADPH/ATP supply from the electron transport chain with metabolic consumption by the CBB cycle.

Kinetic coupling—encompassing rate constants, binding affinities, and the effective concentrations of network components—can strongly shape redox-network behavior and can even allow canonical thioredoxins such as Trx-*f* to promote oxidation under particular conditions, depending on how electrons are routed to competing sinks (12–14). At the same time, *E*_m_ values impose fundamental thermodynamic constraints by setting the driving force and feasible directionality of electron transfer at equilibrium. In practice, physiological redox outcomes reflect the interplay of these two layers. Consistent with this combined view, Yoshida et al. showed that Trx-*f* is capable of oxidizing CBB cycle enzymes, yet an oxidizing Trx-like factor with a higher (less negative) *E*_m_, TrxL2, oxidizes the same targets more efficiently, and the TrxL2/2-Cys peroxiredoxin cascade provides a strong oxidative pull toward H_2_O_2_ (11). The scope of this network has expanded considerably in recent years. For example, Zimmer et al. used *E*_m_-anchored analysis to probe whether targets are controlled near thermodynamic equilibrium with Trx or instead subject to kinetically constrained regulation during photosynthetic induction (15). Yet, despite this conceptual progress, a quantitative energetic map for most native targets is missing because *E*_m_ values are known for only a small fraction of disulfide sites.

In general, determining *E*_m_ has required purified proteins equilibrated in buffers of defined potential. That approach is powerful but labor-intensive, and expression of properly folded, disulfide-containing proteins is often challenging (16, 17). Accordingly, *E*_m_ values have been reported for only a limited number of proteins, and, to our knowledge, proteome-scale determination of disulfide *E*_m_ values has not been described in any organism, including cyanobacteria. In addition, measurements sometimes rely on mutant constructs (e.g., replacing non-participating cysteines with serine) or on isolated domains to simplify the system [e.g., for cyanobacterial EF-G or for chloroplast cascades (18, 19)]. Such modifications can perturb the native *E*_m_, raising concerns about how faithfully they report the in-cell energetics.

To overcome these limitations, we set out to quantify *E*_m_ proteome-wide directly from native lysates of *Synechocystis* sp. PCC 6803 (hereafter *Synechocystis*). Our workflow equilibrates native lysates across defined dithiothreitol (DTT) redox buffers and then uses differential iodoTMT labeling for reduced thiols to determine the absolute reduced/total fraction for each peptide. Fitting these values to the Nernst equation (with an allowance for a non-responsive fraction) yields site-specific *E*_m_ values without protein purification. Finally, by combining the resulting *E*_m_ with absolute in vivo redox ratios measured under light and dark, we compute intracellular (operational) potentials *E* for selected regulatory disulfides, enabling us to ask whether key CBB control points are at equilibrium with TrxA or held away from equilibrium by oxidative partners in vivo.

## Results

### A Proteome-Wide Workflow to Determine *E*_m_ from Native Lysates.

**Fig. 1.**
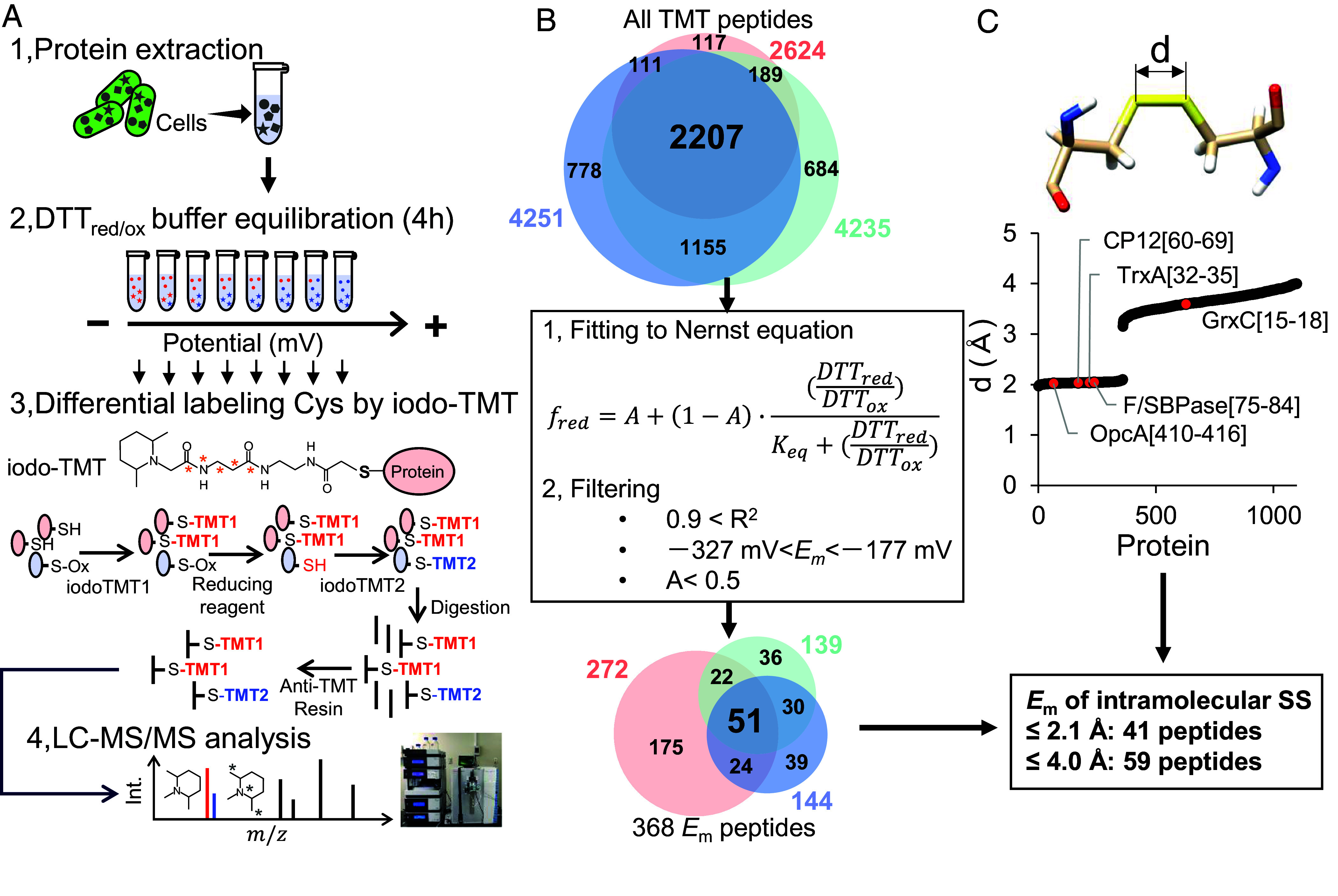
Workflow and coverage of proteome-wide midpoint potential (*E*_m_) determination in *Synechocystis* sp. PCC 6803. (*A*) Schematic of the lysate-based redox proteomics workflow. Native proteins extracted from *Synechocystis* are equilibrated for 4 h in a series of eight reduced and oxidized dithiothreitol (DTT) buffers spanning −327 to −177 mV, followed by differential labeling of reduced and oxidized cysteines with two sixplex iodoTMT reagents, tryptic digestion, anti-TMT immunoenrichment, and LC–MS/MS analysis. The exact concentration and reporter-channel assignments used in each of the three independent experiments are listed in *SI Appendix,* Table S1. (*B*) Venn diagram (*Top*) showing the overlap of all Cys-containing TMT-labeled peptides quantified across three independent experiments (total >2,000 peptides per replicate). Potential-response curves were fitted to an extended Nernst equation including a nonresponsive fraction A, and peptides were filtered using the criteria R^2^ > 0.9, −327 mV < *E*_m_ < −177 mV, and A < 0.5, with redundant peptide forms removed. The Venn diagram (*Bottom*) summarizes the resulting 368 unique *E*_m_-defined peptides, of which 51 were reproducibly quantified in all three experiments. (*C*) Distribution of nearest sulfur–sulfur (S–S) distances *d* for all cysteines in the *Synechocystis* AlphaFold proteome, used to classify intramolecular disulfides. Using a strict S–S threshold of ≤2.1 Å yields 41 *E*_m_-defined peptides assigned to intramolecular disulfides; relaxing the threshold to ≤4.0 Å increases this number to 59 peptides (Dataset S2).

To quantify disulfide *E*_m_ at scale, we equilibrated native lysates of *Synechocystis* for 4 h in eight DTT red/ox buffers spanning −327 to −177 mV and then read out the reduced/total (Red/Total) fraction of each cysteine by differential iodoTMT labeling (Fig. 1*A* and *SI* *Appendix*, Table S1). The workflow follows and generalizes differential TMT strategies with the sequence: i) label preexisting reduced thiols with a first sixplex iodoTMT reagent set (label 1), ii) reduce disulfides with tris(2-carboxyethyl)phosphine (TCEP), iii) label the newly formed thiols with a second sixplex iodoTMT reagent set (label 2), iv) tryptic digestion and anti-TMT resin enrichment, and v) LC–MS/MS quantification. Plotting Red/Total against the buffer potential and fitting to the Nernst function yields site-specific *E*_m_ values. By construction, *E*_m_ can be derived directly from Red/Total only for intramolecular disulfide/dithiol couples.

Across three independent experiments, >2,000 cysteine-containing peptides were detected per replicate (Fig. 1*B*). Dataset S1 reports the Red/Total values for peptides that were detected in all eight potential samples within each replicate. A fraction of potential–response curves showed incomplete oxidation at the most positive potentials, a behavior also reported for purified proteins (cause unknown) (20). To accommodate this, we fitted an extended Nernst equation that includes a nonresponsive fraction, A, and then applied stringent quality criteria: R^2^ > 0.9, −327 mV < *E*_m_ < −177 mV, and A < 0.5, followed by removal of redundant peptides (missed cleavages, Met-oxidized forms). This yielded 368 accepted *E*_m_ values in total, 51 of which were reproduced across all three experiments (Dataset S2). To assess which of these sites are likely to represent intramolecular disulfides, we calculated, for every cysteine in *Synechocystis*, the distance to the nearest cysteine sulfur atom across the entire AlphaFold proteome. Previous structural analyses have shown that the mean S–S distance of protein disulfide bonds is typically ~2.04 to 2.05 Å and that a threshold of ~2.5 Å is commonly used for geometric identification of disulfides (21–23). Consistent with this, the distribution of nearest-neighbor S–S distance was bimodal with a gap at ~2.10 Å and ~3.15 Å, enabling a simple discrimination between bonded and nonbonded pairs in predicted structures (Fig. 1*C*). As reference points, we highlighted known disulfide-forming proteins from *Synechocystis* in Fig. 1*C*, whose AlphaFold S–S distances fall within or near the bonded cluster. In the *Synechocystis* AlphaFold set, we identified 358 intramolecular disulfides with S–S distances 1.97 to 2.10 Å. Of these, 41 overlapped with sites for which we determined *E*_m_ values (Dataset S2). Notably, intramolecular disulfides can also occur at S–S distances >3 Å in AlphaFold models; for example, the Cys15–Cys18 pair of glutaredoxin has an AlphaFold S–S distance of 3.59 Å yet forms a reversible disulfide in vitro (24). We therefore considered cysteine pairs with S–S ≤4 Å as potential disulfides, which increases the number of *E*_m_-defined cysteine sites assignable to intramolecular disulfides from 41 to 59 (Dataset S2). Taking these into account, *E*_m_-defined Cys peptides can be classified into eight categories based on the number of cysteines they contain and the presence or absence of an intramolecular disulfide (*SI Appendix,* Fig. S1), among which categories #2, #4, #5, #6, and #8 correspond to intramolecular disulfide types. For peptides in the remaining categories, whether they form intramolecular disulfides remains unresolved (Dataset S2). The dataset thus provides a robust, lysate-based atlas of disulfide *E*_m_ values against which regulatory hierarchies can be evaluated.

### Potential Responses and the Distribution of *E*_m_ Values.

**Fig. 2.**
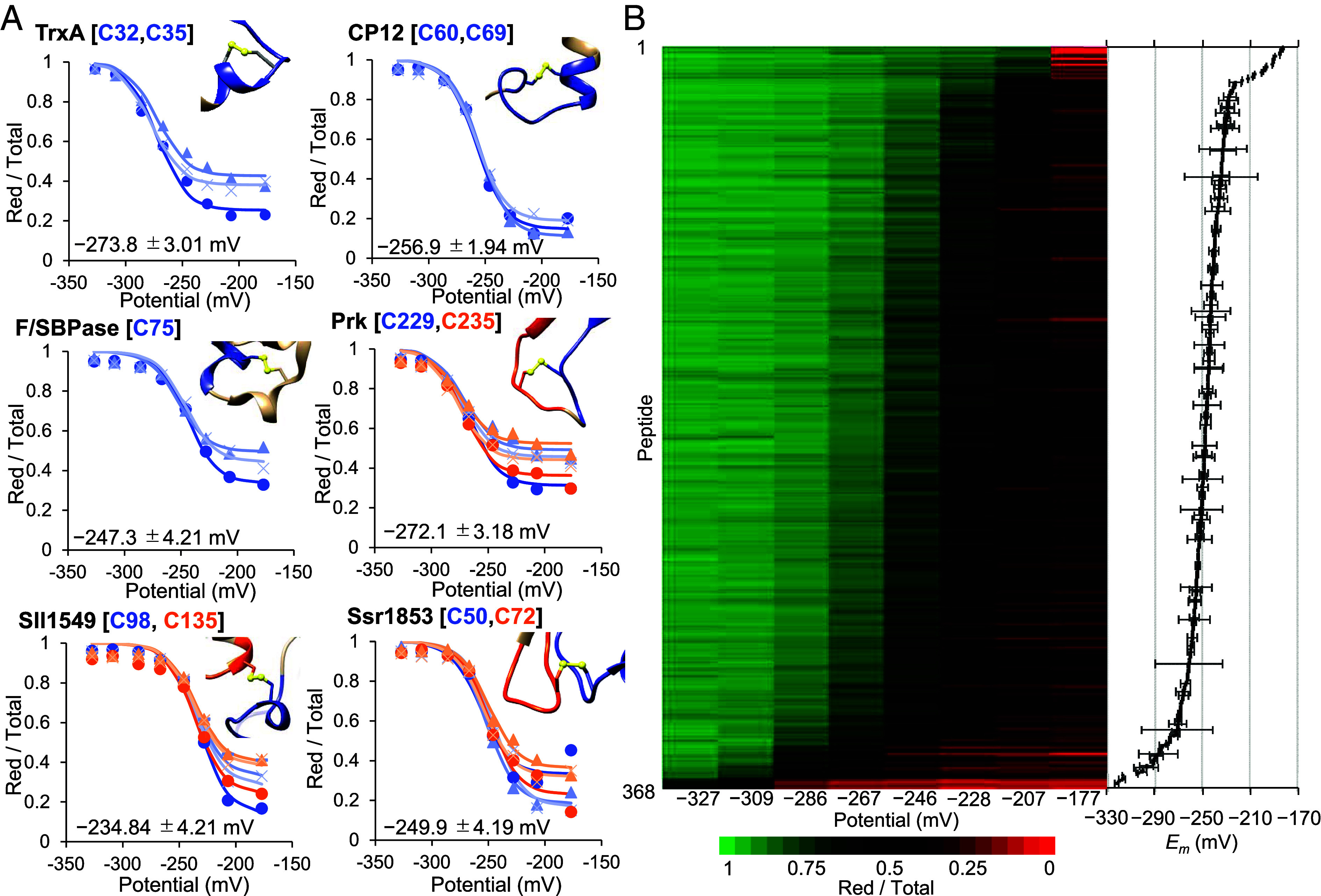
Nernst behavior and *E*_m_ distribution of regulatory and nonregulatory disulfides. (*A*) Representative Nernst plots for selected regulatory and structural disulfides. Reduced fractions (Red/Total) are shown for TrxA [C32–C35], CP12 [C60–C69], F/SBPase [C75], PRK [C229–C235], Sll1549 [C98–C135], and Ssr1853 [C50–C72] across the eight DTT buffer potentials. Solid lines indicate fits to the extended Nernst equation; insets show the corresponding AlphaFold structures with the disulfide highlighted. For TrxA, CP12, and F/SBPase, three fitted curves are shown, corresponding to three independent experiments. For PRK, Sll1549, and Ssr1853, six fitted curves are shown because the two cysteines forming the disulfide were detected as separate peptides, yielding two independent Nernst plots per experiment. Mean *E*_m_ values ± SD are indicated for each site (n = 3, independent experiments). (*B*) Heatmap of reduced fractions for all 368 *E*_m_-defined peptides ordered by their fitted *E*_m_ (*Left*) and histogram of *E*_m_ distribution (*Right*).

Disulfides known or proposed to participate in redox regulation in cyanobacteria—TrxA, F/SBPase, CP12, and PRK—were robustly detected (Fig. 2*A*). For TrxA (C32–C35) and CP12 (C60–C69), the two cysteines appeared on the same tryptic peptide (category #4), whereas for PRK the regulatory C-terminal disulfide (C229–C235) was cleaved into two peptides because an intervening Arg separates the cysteines (category #2). The two PRK peptides yielded essentially identical *E*_m_ values (S.D. 3.18 mV), supporting the precision of site assignment. Similarly, AlphaFold-supported intramolecular disulfides in Sll1549 (a putative protein serine/threonine phosphatase) and Ssr1853 (uncharacterized protein) were quantified as separate peptides yet gave closely matching *E*_m_ values (S.D. 4.21 mV and 4.19 mV, respectively). Representative fits returned −247.3 ± 4.2 mV for F/SBPase, −256.9 ± 1.9 mV for the CP12 C-terminal disulfide, and −272.1 ± 3.18 mV for PRK (Fig. 2*A*). Proteome-wide, potential-response heatmaps revealed a dominant *E*_m_ band around −250 ± 10 mV, accounting for 55.5% of quantified sites (Fig. 2*B*).

### Agreement Between Proteome-Wide *E*_m_ Values and Purified-Protein Measurements.

**Fig. 3.**
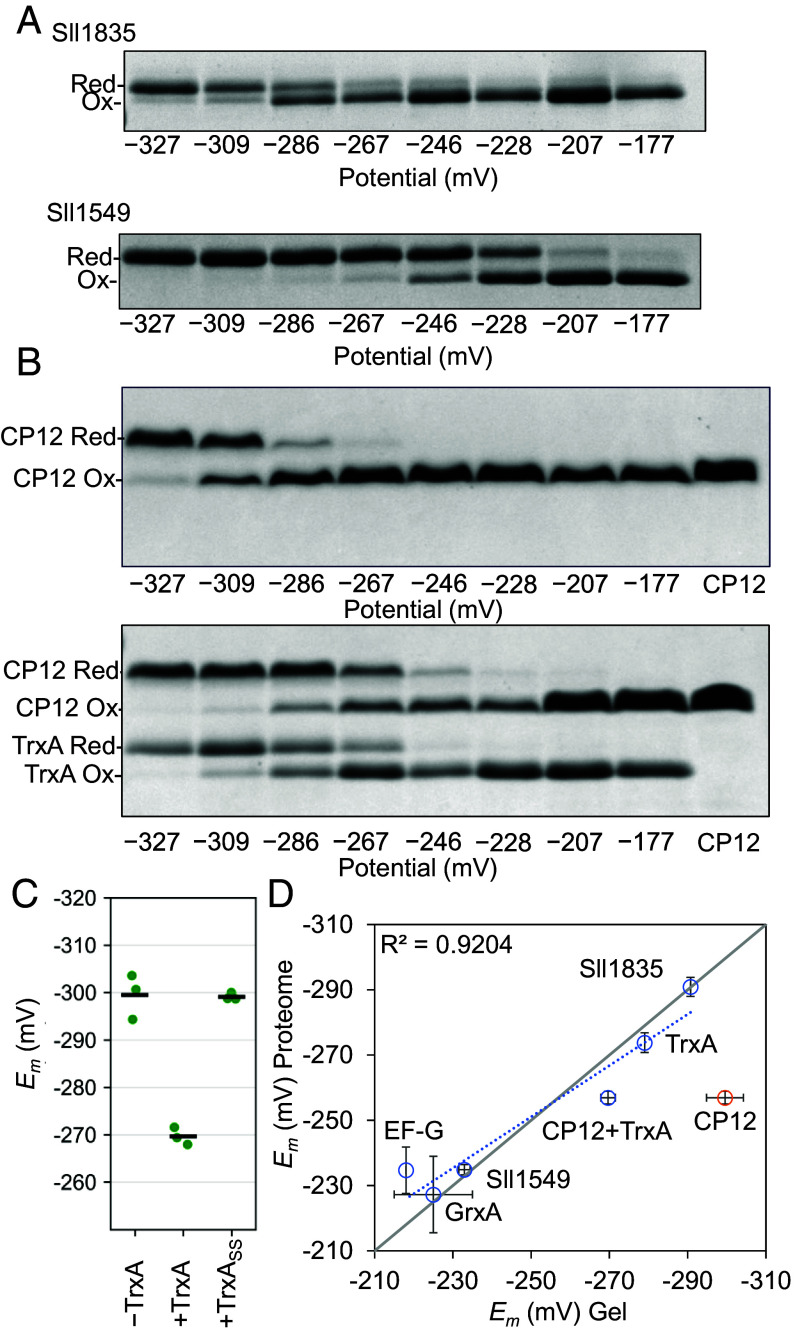
Benchmarking lysate-derived *E*_m_ values against purified proteins and context dependence of CP12. (*A*) Redox titrations of purified Sll1835 and truncated Sll1549 (N-terminal transmembrane region removed, Δ1 to 45) monitored by nonreducing SDS–PAGE after maleimide labeling across the eight DTT buffer potentials. Red and Ox denote reduced and oxidized forms, respectively. (*B*) Redox titration of CP12_SSCC_ in the absence (*Top*) or presence (*Bottom*) of TrxA under identical buffer conditions. (*C*) Comparison of *E*_m_ values for CP12_SSCC_ determined from purified protein titrations without (−TrxA) or with TrxA (+TrxA). Including a catalytically inactive TrxA variant in which the active-site cysteines (C32 and C35) were replaced by serines (TrxA_SS_) did not shift the apparent *E*_m_ of CP12_SSCC_ (see also *SI Appendix,* Fig. S2). Each point represents one independent experiment (n = 3), with horizontal bars indicating means. (*D*) Correlation between *E*_m_ values obtained from the proteome-wide lysate workflow (y-axis) and from purified protein titrations (x-axis) for TrxA, GrxA, EF-G, Sll1549, Sll1835, and CP12 (with and without TrxA). The solid line represents the line of identity; the dashed line indicates the best-fit regression (R^2^ = 0.9204).

To benchmark accuracy, we determined *E*_m_ values for purified proteins chosen to contain no other cysteines beyond the disulfide of interest. Redox titrations gave −290.4 ± 0.4 mV for Sll1835 (uncharacterized protein) and −232.9 ± 1.4 mV for Sll1549, in close agreement with the proteome-wide values (−290.9 ± 2.9 mV and −234.8 ± 1.6 mV, respectively) (Fig. 3*A*). We next focused on the CBB cycle regulator CP12 (Ssl3364), for which our proteome-wide analysis yielded the C-terminal disulfide *E*_m_ (−256.9 ± 1.9 mV). A construct with the N-terminal Cys pair replaced by Ser (CP12_SSCC_) gave a substantially more negative *E*_m_ (−299.5 ± 4.7 mV) when titrated as a purified protein (Fig. 3*B*). Notably, including TrxA during titration shifted the apparent *E*_m_ to −269.7 ± 1.8 mV, closer to the proteome-wide value (Fig. 3*C*). To test whether this shift could be explained by TrxA binding alone, we repeated the titration in the presence of a catalytically inactive TrxA variant in which the active-site cysteines (C32 and C35) were replaced with serines (TrxA_SS_). Under these conditions, CP12_SSCC_ showed no measurable *E*_m_ shift relative to CP12_SSCC_ alone (Fig. 3*C* and *SI Appendix,* Fig. S2). This result indicates that catalytic activity, rather than binding alone, is required for the TrxA-dependent shift in the apparent *E*_m_. Although the lysate-based workflow used here operates at concentrations far below those in vivo and therefore cannot fully preserve all intracellular interactions, the CP12 results suggest that inclusion of endogenous redox partners can mitigate underequilibration artifacts that may arise in isolated-protein assays. Incorporating previously reported *E*_m_ values for TrxA (18), GrxA (24), and EF-G (18) with our purified proteins revealed a strong overall correlation between purified-protein and proteome-wide *E*_m_ (R^2^ = 0.92; Fig. 3*D*).

### Redox Cascade from TrxA and *E*_m_ Hierarchy.

In redox cascades, electron flow generally proceeds from sites with lower (more negative) *E*_m_ to those with higher (more positive) *E*_m_, providing a thermodynamic ordering of donor–acceptor pairs. We therefore examined whether the CBB cycle control disulfides of *Synechocystis* follow such a cascade. Cyanobacteria are known to harbor reversible regulatory disulfides in F/SBPase, CP12, and PRK (7). Among them, F/SBPase has been shown to be reduced by TrxA in *Synechocystis* (25). For CP12 and PRK, Trx-dependent reduction has been reported in other phototrophs (26, 27), but not previously demonstrated in *Synechocystis*.

**Fig. 4.**
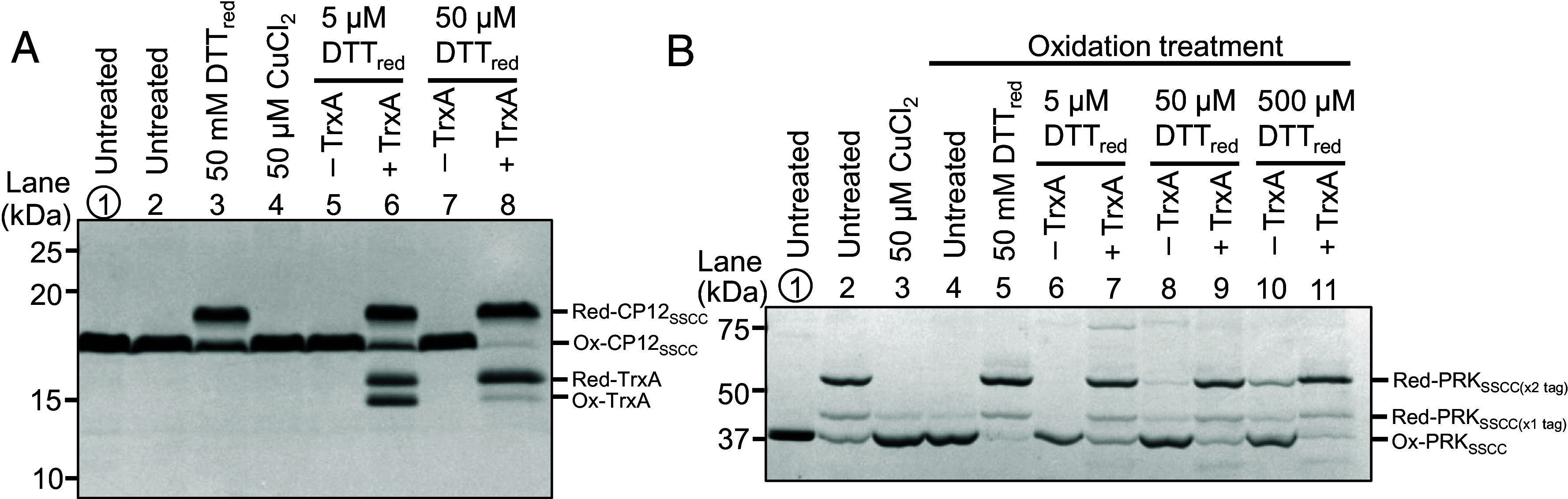
TrxA-catalyzed reduction of CP12 and PRK. (*A*) Nonreducing SDS–PAGE of purified CP12_SSCC_ after various redox treatments. Lanes show untreated protein, fully reduced protein (50 mM DTTred), oxidized protein (50 µM CuCl_2_), and samples treated with low concentrations of DTTred (5 or 50 µM) in the absence or presence of TrxA. CP12_SSCC_ is only efficiently reduced when TrxA is present, indicating TrxA-catalyzed electron transfer. (*B*) Nonreducing SDS–PAGE of PRK_SSCC_ after preoxidation with CuCl_2_ and subsequent incubation with 50 mM or 5 to 500 µM DTTred with or without TrxA. Similar to CP12, TrxA enables reduction of the C-terminal regulatory disulfide. Major redox species are indicated on the *Right*. In addition to the fully tagged reduced form, a singly tagged reduced PRK_SSCC_ species was observed, likely reflecting incomplete double labeling by the bulky maleimide tag. Representative gels are shown from n = 3 independent experiments.

We therefore tested whether *Synechocystis* TrxA can catalyze reduction of these proteins. CP12 in *Synechocystis* contains two cysteine pairs, one in the N-terminal region and the other in the C-terminal region. The peptide containing the N-terminal cysteine pair was not detected in our redox proteomics workflow for unknown reasons, and therefore only the C-terminal pair was quantified in the proteome-wide *E*_m_ analysis. This focus is also biologically relevant because previous studies in *Synechocystis* showed that the C-terminal cysteine pair has a stronger impact than the N-terminal pair on glucose utilization and suppression of NADPH oxidation (28, 29). We therefore first analyzed the C-terminal pair in detail. Purified CP12_SSCC_ (with the N-terminal cysteines replaced) migrated predominantly in an oxidized form (Fig. 4*A*, lanes 1 to 2). Addition of 50 mM DTT reduced it only partially after 30 min (lane 3). Incubation with 5 or 50 μM DTT left the protein oxidized (lanes 5, 7). In contrast, TrxA strongly promoted reduction at these low DTT concentrations (lanes 6, 8). These observations indicate that TrxA catalyzes reduction of the C-terminal disulfide and likely accelerates equilibration of CP12_SSCC_ with the DTT redox buffer, which is consistent with the titration results described above (*SI Appendix,* Fig. S2). We further examined whether the N-terminal cysteine pair can also be reduced by TrxA using a complementary mutant construct in which the C-terminal pair was replaced (CP12_CCSS_). Under the same assay conditions, the N-terminal pair also showed TrxA-dependent reduction (*SI Appendix,* Fig. S3), suggesting that both cysteine pairs of CP12 can, in principle, be reduced by TrxA.

For PRK, which forms two intramolecular disulfides, the N-terminal pair was only minimally reduced by TrxA in the complementary mutant construct (PRK_CCSS_, *SI Appendix,* Fig. S4*A*), consistent with the conclusion from *Anabaena* sp. PCC 7120 PRK that thioredoxin preferentially targets the C-terminal cysteine pair (27). The N-terminal cysteine pair also showed potential-dependent redox behavior, but its fit did not satisfy our acceptance criterion for the nonresponsive fraction (A < 0.5); accordingly, it was excluded from the main *E*_m_ dataset, although its fitted *E*_m_ was −264.1 ± 10.4 mV as a reference value (*SI Appendix,* Fig. S4*B*). On the contrary, our proteome-wide analysis quantified *E*_m_ of the C-terminal pair. We therefore focused the main functional analysis on the C-terminal pair. A construct in which the N-terminal Cys pair was replaced (PRK_SSCC_) was partially oxidized with CuCl_2_ (Fig. 4*B*, lanes 3 to 4). The oxidized protein was reduced by 50 mM DTT (lane 5) but not effectively by 5, 50, or 500 μM DTT alone (lanes 6, 8, 10). TrxA enabled reduction under these low-reductant conditions (lanes 7, 9, 11), confirming that the C-terminal regulatory disulfide of PRK is a TrxA target in *Synechocystis*. In PRK_SSCC_, an additional band corresponding to a singly tagged reduced form was also observed, likely because the bulky thiol-reactive tag did not fully modify both reduced cysteines as reported previously (30, 31).

Together, these assays establish that in *Synechocystis*, TrxA reduces both cysteine pairs of CP12 and C-terminal cysteine pair of PRK, consistent with their placement in the TrxA-centered cascade. Thermodynamically, the *E*_m_ of TrxA is more negative than that of the C-terminal cysteine pair of CP12, whereas it is similar to that of the C-terminal cysteine pair of PRK. This indicates that the redox gap between TrxA and its targets differs among control points of the CBB cycle.

### Intracellular Operational Potentials Under Light and Dark.

Although the *E*_m_ hierarchy described above defines the thermodynamic order of potential electron transfer, it does not by itself reveal the operational state in vivo—namely, which disulfides are held at equilibrium with TrxA and which are biased away from it. To address this, we computed intracellular operational potentials *E* by combining site-specific *E*_m_ values with absolute reduced/total ratios determined in cells under light and dark conditions.

**Fig. 5.**
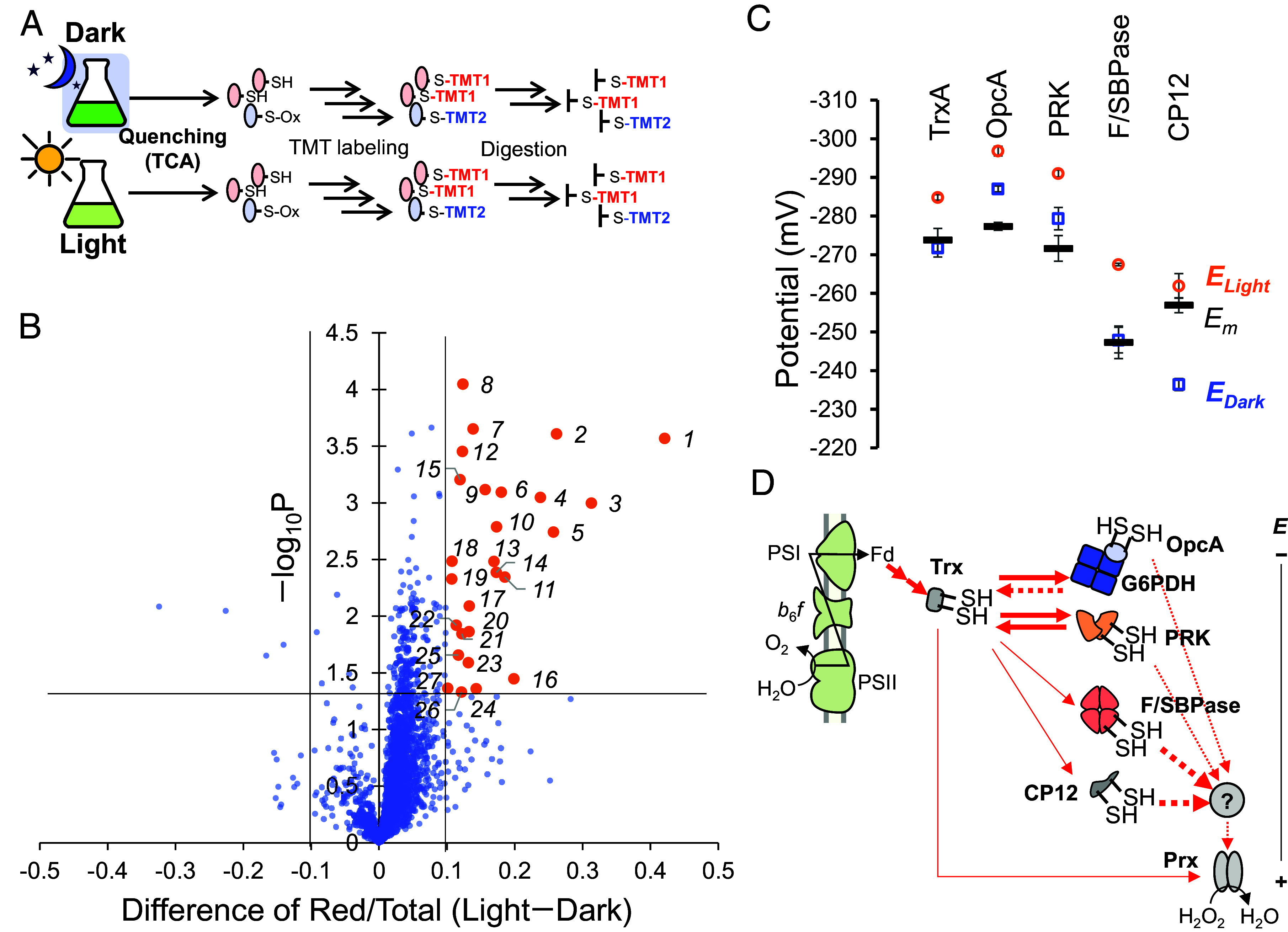
Light–dark control of cysteine redox state and organization of the TrxA-centered redox hierarchy. (*A*) Scheme of the iodoTMT-based workflow used to quantify absolute reduced/total fractions in vivo under dark and light conditions. Cultures are quenched directly with trichloroacetic acid (TCA), followed by sequential iodoTMT labeling, digestion, and LC–MS/MS. (*B*) Volcano plot of light-induced changes in reduced fraction (Light–Dark) versus −log_10_P for all quantified cysteine-containing peptide entries in Dataset S3. Peptides that become significantly more reduced in the light (≥10% and *P* < 0.05) are shown in orange dots and labeled by rank number. The corresponding identities are listed in Table 1. (*C*) Comparison of *E*_m_ (black) and intracellular operational potentials E_Light_ (orange) and E_Dark_ (blue) for TrxA, OpcA, PRK, F/SBPase, and CP12. Error bars denote SD (n = 3). PRK operates near the TrxA potential, whereas F/SBPase and CP12 are held at substantially more oxidized potentials. (*D*) Conceptual model of the TrxA-centered redox network in *Synechocystis*, highlighting electron flow from photosystem (PS) I via ferredoxin (Fd) and TrxA to PRK, F/SBPase, and CP12, the peroxiredoxin (Prx)-linked branch connecting TrxA to peroxide sinks, and additional unresolved oxidative factor(s) inferred for F/SBPase and CP12. OpcA/G6PDH is shown as a distinct oxidative node.

A previous work used redox proteomics to report relative light/dark changes in thiol oxidation across the *Synechocystis* proteome, revealing that many cysteines are redox-responsive to illumination (32). However, their approach relied on 3-(3,4-dichlorophenyl)-1,1-dimethylurea (DCMU)-treated samples as a reference and therefore did not yield absolute red/total fractions, making it impossible to compute *E* for specific disulfide–dithiol pairs. In contrast, by applying iodoTMT-based absolute quantification, we obtained absolute red/total ratios under both conditions and converted them to *E* values through the Nernst equation. This provides a direct energetic readout of each site in vivo (Fig. 5*A*).

Across the proteome (Dataset S3), 27 peptides (22 proteins) were ≥10% more reduced in the light (Fig. 5*B* and Table 1). Many of these were CBB cycle control nodes, allowing us to compare their operational potentials with TrxA. The results showed that C-terminal Cys pair of PRK was maintained slightly more negative than TrxA (Δ*E* ≈ −6.2 mV in light; −7.6 mV in dark), consistent with near-equilibrium behavior. By contrast, F/SBPase and C-terminal Cys pair of CP12 were held substantially more oxidized than TrxA (F/SBPase: +17.4 mV light, +23.9 mV dark; CP12: +22.9 mV light, +35.4 mV dark), demonstrating nonequilibrium organization with a strong oxidative bias (Fig. 5*C*).

**Table 1.** Light-responsive cysteine-containing peptide entries identified by absolute red/total quantification

						Red/Total (%)	
Rank (Fig. 5*B*)	Protein name	Gene ID	Gene	Peptide detected	Cys	Light	Dark
1	CP12	*ssl3364*	*cp12*	59 to 70	60, 69	59.4	17.2
2	F/SBPase	*slr2094*	*glpX, fbpI*	53 to 77 (Oxidation M65)	75	77.1	50.9
3	F/SBPase	*slr2094*	*glpX, fbpI*	53 to 77	75	82.4	51.1
4	TrxA	*slr0623*	*trxA*	14 to 36	32, 35	69.9	46.1
5	F/SBPase	*slr2094*	*glpX, fbpI*	61 to 77	75	79.4	53.7
6	PedR	*ssl0564*	*pedR*	72 to 89	73, 79, 80	51.6	33.6
7	OxPP cycle protein OpcA	*slr1734*	*opcA*	396 to 418	405, 410, 416	81.6	67.7
8	OxPP cycle protein OpcA	*slr1734*	*opcA*	197 to 211	204, 205	71.9	59.5
9	SbtB	*slr1513*	*sbtB*	83 to 110	94, 105, 110	39.1	23.4
10	UDP-N-acetylmuramate--L-alanine ligase	*slr1423*	*murC*	310 to 337	312	84.2	66.9
11	Sll0149 protein	*sll0149*		123 to 132	128	80.1	61.6
12	OxPP cycle protein OpcA	*slr1734*	*opcA*	171 to 196	175, 192	82.9	70.6
13	3-oxoacyl-ACP synthase 2	*sll1069*	*fabF*	158 to 179	162, 167	75.1	58.2
14	PRK	*sll1525*	*prk*	211 to 231	229	81.5	64.3
15	Acetolactate synthase	*sll1981*	*ilvB*	541 to 550	546	82.4	70.4
16	Sll0414 protein	*sll0414*		94 to 113	106	72.3	52.5
17	Cell division cycle protein	*slr0374*		260 to 271	269	91.6	78.3
18	Sll1466 protein	*sll1466*		353 to 376	367	80.0	69.3
19	Sll0585 protein	*sll0585*		104 to 120	109	86.7	76.0
20	PRK	*sll1525*	*prk*	233 to 241	235	78.3	65.0
21	Long-chain acyl-ACP reductase	*sll0209*		59 to 72	63	88.4	76.2
22	Slr1194 protein	*slr1194*		112 to 129	123	79.0	67.6
23	4-alpha-glucanotransferase	*sll1676*	*malQ*	6 to 19	6	85.0	71.8
24	Slr1732 protein	*slr1732*		73 to 90	84	79.9	65.6
25	Glycolate oxidase subunit GlcD	*sll0404*	*glcD*	413 to 430	413	78.5	66.9
26	Sll1336 protein	*sll1336*		50 to 61	51	83.7	71.5
27	UDP-ManNAcA transferase	*slr1271*	*rffM*	90 to 101	90	85.9	75.8

Peptide-level entries that were significantly more reduced in the light than in the dark in the in vivo iodoTMT-based redox analysis. Rank numbers correspond to the highlighted entries in Fig. 5*B*. For each entry, the table lists the protein name, locus/gene annotation, peptide coordinates, quantified cysteine position(s), and mean reduced/total values under light and dark conditions. Distinct rows from the same protein represent independently quantified peptide/site-level entries, including overlapping peptides, differently modified peptide forms, or separate cysteines detected on different peptides. Abbreviations: ACP, acyl carrier protein; F/SBPase, fructose 1,6-bisphosphatase/sedoheptulose 1,7-bisphosphatase; OxPP, oxidative pentose phosphate; PRK, phosphoribulokinase; TrxA, thioredoxin A; UDP-ManNAcA, UDP-N-acetyl-D-mannosaminuronic acid.

To formalize these classifications, we quantified the degree of thermodynamic equilibration between each site and TrxA using the Equilibration Index (EI), which integrates both experimental uncertainty and a defined tolerance window. The mathematical definition of EI and its calculation procedure are described in *Materials and Methods*. In brief, an EI value of 1 indicates that the site’s potential is statistically indistinguishable from TrxA (i.e., thermodynamic equilibrium), whereas EI gradually decreases as the potential difference exceeds the confidence range, approaching zero for clearly nonequilibrium states. We interpreted EI > 0.5 as indicative of near-equilibrium conditions. By this definition, PRK exhibited EI > 0.5 in both light and dark, confirming that it is maintained close to TrxA’s potential. In contrast, F/SBPase and CP12 had EI = 0, indicating nonequilibrium organization where oxidative electron outputs dominate over TrxA reduction (Fig. 5*D*). Proteome-wide, among the 329 peptides for which *E* could be calculated, 69 peptides exhibited EI > 0.5 under both conditions, indicating that they remain near-equilibrium with TrxA, whereas the majority operate at potentials significantly different from TrxA, reflecting nonequilibrium states (Dataset S4).

Thus, our absolute quantification reveals that although many disulfide switches are responsive to light/dark, only a subset operate at or near equilibrium with TrxA. The combination of proteome-wide *E*_m_, absolute *E*, and EI analysis establishes that the *Synechocystis* redox network is organized as a nonequilibrium hierarchy, in which different targets of TrxA are differentially biased toward reductive or oxidative control.

## Discussion

We established a lysate-based redox-proteomics workflow that determines disulfide *E*_m_ proteome-wide in *Synechocystis* and validated the values against purified proteins. An important advantage of this design is that titrations are performed in native lysates, so even when the physiological redox partner of a given protein is unknown, endogenous factors retained in the extract can contribute to the apparent equilibrium reached under the imposed potential series, as illustrated by the TrxA-dependent convergence observed for CP12. However, this advantage must be interpreted together with an important limitation. The proteome-wide titrations were carried out under strongly diluted conditions (200 µg protein in 400 µL), far below the macromolecular crowding expected in bacterial cytoplasm (33), because equilibration at near-physiological protein concentrations would allow protein thiols to measurably perturb the intended DTT_red_/DTT_ox_ ratio, especially under the more oxidizing conditions, and thereby compromise a well-defined potential series (See also *Materials and Methods*). We therefore chose diluted lysates together with repeated buffer exchange to preserve reproducible buffer potentials. Accordingly, the present workflow yields *E*_m_ values for native proteins under diluted-lysate conditions and captures interaction effects only to the extent that they persist at those effective concentrations. Thus, it should not be viewed as a full reconstruction of in vivo binding, competition, or electron flux. This caveat is particularly relevant for thioredoxins, which can function not only as electron carriers but also as binding partners partly independently of electron transfer (34). Future extensions could therefore combine defined redox buffers with controlled supplementation of key transmitters such as TrxA to estimated cytosolic concentrations, or implement continuous dialysis/buffer-refresh strategies that permit equilibration at much higher protein concentrations. Such developments, conceptually analogous to recent in vitro redox-network reconstitutions assembled near in vivo component concentrations (35), should help bridge proteome-wide *E*_m_ mapping to experimentally grounded models of dynamic redox-network behavior.

Another advantage is that the workflow yields *E*_m_ values for native proteins, without requiring cysteine substitutions or domain truncations. The largest discrepancy between conventional purified-protein measurements and our lysate-based *E*_m_ values occurred for EF-G (Fig. 3*D*), likely because the previous *E*_m_ determination relied on a multicysteine mutant (C68S/C388S/C676S) (18). A similar caveat may apply to CP12, for which the purified-protein titration was performed with the N-terminal cysteine pair replaced, and this substitution may have contributed to the remaining difference between the purified-protein and lysate-based *E*_m_ values (Fig. 3*D*). In contrast, our approach infers *E*_m_ from the unmodified native protein, highlighting the importance of investigating native sequences whenever possible.

Network interpretation of *E*_m_ together with intracellular operational potentials *E* reveals that the *Synechocystis* TrxA cascade is not uniformly at equilibrium with its targets (Fig. 5 *C* and *D*). The *E*_m_ of *Synechocystis* TrxA lies within the range reported for canonical photosynthetic thioredoxins, including cyanobacterial Trx-*m*-type proteins and chloroplast Trx isoforms (36–38). Thus, the key implication of our data is not that *Synechocystis* uses an unusual thioredoxin, but that different target proteins are maintained at markedly different in vivo redox states relative to TrxA. Even among canonical CBB control points, the balance between reductive (TrxA) and oxidative influences is target-specific. PRK behaved as a near-equilibrium TrxA target, consistent with direct bidirectional exchange with TrxA. In plant chloroplasts, canonical Trx-*f* can reduce CBB enzymes but can also promote their oxidation under certain conditions, whereas more specialized oxidative pathways involving Trx-like proteins such as TrxL2 and atypical cysteine histidine-rich Trxs (ACHT) together with 2-Cys Prx further bias CBB cycle enzymes toward oxidation (11–13, 39, 40). Because PRK was maintained close to the TrxA potential, oxidation of TrxA would be expected to propagate readily to PRK. Consistent with this idea, we confirmed that oxidized TrxA oxidizes reduced PRK_SSCC_ (*SI Appendix,* Fig. S5). In *Synechocystis*, TrxA is also known to donate electrons to peroxiredoxins (41), providing a route by which reducing equivalents can ultimately be coupled to peroxide sinks. These observations make a TrxA-linked oxidative route for the C-terminal PRK disulfide plausible (Fig. 5*D*). By contrast, F/SBPase and CP12 (the C-terminal cysteine pair) operated at substantially more oxidizing potentials than TrxA, implying additional oxidative influences beyond simple equilibration with TrxA. Cyanobacteria do not appear to encode clear counterparts of the chloroplast TrxL2 and ACHT oxidative branches (11, 39, 40, 42), so the factors that bias F/SBPase and CP12 toward oxidation remain unresolved. Defining these branches, including possible coupling to Prx- and H_2_O_2_-dependent pathways, will be an important next step.

Absolute red/total quantification in light and dark identified 27 peptides (22 proteins) that became ≥10% more reduced in the light (Fig. 5*B* and Table 1), with CBB components prominently represented. For PRK, only the C-terminal pair (C229–C235) responded to light/dark, exactly matching a report from other cyanobacteria (e.g., *Anabaena*) (27), while the N-terminal pair remained unresponsive (Fig. 5*B*). Beyond the CBB cycle, however, the responsive set suggests that light-dependent thiol regulation extends to carbon acquisition/signaling, transcriptional regulation, and catabolic rerouting of reducing power. SbtB (Slr1513), a PII-like regulator linked to bicarbonate uptake and metabolic signaling, became more oxidized in the dark. Because SbtB is known to integrate metabolic and redox cues in the control of the bicarbonate transporter SbtA, this result supports the idea that the Ci-acquisition module is directly engaged by thiol-based redox regulation in vivo (43). PedR (Ssl0564), a redox-responsive transcriptional regulator, showed a similar dark-state oxidation, consistent with coupling between photosynthetic redox poise and transcriptional acclimation (44). OpcA, the redox sensor that controls activation of G6PDH in the oxidative pentose phosphate pathway, also shifted toward oxidation in darkness, consistent with enhanced oxidative carbohydrate catabolism when photosynthetic reducing input ceases (45). Taken together, these examples indicate that the light/dark-responsive disulfides detected here extend beyond core CBB enzymes to proteins involved in Ci uptake control, transcriptional regulation, and redox balancing. This broader pattern is consistent with the view that thiol-redox regulation in cyanobacteria extends well beyond classical CBB-cycle enzymes (7).

Another notable case is CcmM, a central β-carboxysome scaffold protein previously shown in *Synechococcus elongatus* PCC 7942 to undergo functionally important redox regulation through disulfide formation in small subunit-like (SSUL) modules (46, 47). Although CcmM was not among the strongly light-responsive proteins highlighted in Fig. 5*B*, multiple cysteine-containing peptides from *Synechocystis* CcmM were detected in our proteome-wide dataset. Sequence comparison together with AlphaFold2-based domain interpretation suggests that the *Synechocystis* CcmM contains four SSUL modules, and the redox-active cysteine pairs detected here map to putative SSUL3 (C514–C532; *E*_m_ = −270.9 ± 4.3 mV) and SSUL4 (C639–C657; *E*_m_ = −253.8 ± 2.3 mV) (*SI Appendix,* Fig. S6). This arrangement differs from PCC 7942 CcmM, in which the previously characterized redox-regulated disulfides reside in SSUL1 and SSUL2. Thus, while redox-sensitive SSUL disulfides may be a conserved feature of CcmM-family scaffolds, the domain architecture and placement of the relevant cysteine pairs appear to differ between these cyanobacterial species. The functional consequences of these *Synechocystis* CcmM disulfides for carboxysome organization remain to be determined.

Multiple structural factors have been proposed to shape the *E*_m_ of protein disulfides. In catalytic CXXC motifs such as those of thioredoxin (Trx) and DsbA, the pKa of the cysteine thiol correlates with *E*_m_, consistent with the notion that basicity of the thiolate tunes redox power (48, 49). Beyond ionization, geometric strain within the S–S link and electrostatic stabilization have been implicated as additional contributors (50). Computational efforts using free-energy calculations, MD, and hybrid QM/MM approaches have begun to dissect individual cases (5), but a general decomposition of determinants for native disulfides remains elusive. To estimate which features are most informative, we examined structural correlates of *E*_m_ using AlphaFold models. Among the peptides for which *E*_m_ was determined, 41 mapped to intramolecular disulfides with S–S distances ≤3 Å in the AlphaFold monomer structures (Dataset S2). Restricting the analysis to models with pLDDT ≥ 70 yielded 33 disulfides for correlation analysis. Across these 33 sites, pKa values [estimated by PROPKA (51)] and dihedral strain energy (DSE) [from an empirical dihedral potential (52, 53)] showed no detectable correlation with *E*_m_ (*SI Appendix,* Fig. S7). By contrast, geometric descriptors that report on bond strain—the Cα–Cα distance *c* and the α angle—displayed weak correlations with *E*_m_. Because these geometry parameters are recognized proxies for disulfide strain, the data support the view that disulfide-bond strain contributes measurably to *E*_m_, whereas pKa and simple DSE surrogates—at least as captured by these predictions—seem less informative. In this study, correlations between *E*_m_ and structural parameters could be evaluated at only 33 sites. Future work integrating larger numbers of experimentally determined *E*_m_ values and more intensive model construction will be required to draw broader conclusions.

By construction, our Nernst fits are exact only for two-electron disulfide/dithiol couples in a fixed molecular context, which raises the question of what redox entities are being probed at sites that lack intramolecular disulfides in the AlphaFold monomer. Nondisulfide modifications such as sulfenylation, nitrosylation, or glutathionylation are not titrated as simple disulfide/dithiol couples by DTT and therefore are unlikely to generate the clean Nernst behavior and high-quality fits that passed our filters. In contrast, our dataset almost certainly includes some intermolecular disulfide couples, and for these the intrinsic *E*_m_ and the apparent *E*_m_ reported here need not coincide. The apparent midpoint potential of an intermolecular disulfide depends strongly on protein concentration and shifts to more positive values at lower concentrations (*SI Appendix,* Fig. S8). Under our lysate conditions most proteins are present at ≤1 µM (200 µg total protein in 400 µL), and simple equilibrium modeling of a dimeric couple with an intrinsic *E*_m_ = −470 mV—the most negative intramolecular disulfide value reported to date for proteins (54)—yields an apparent midpoint of −290 mV, which lies within our accepted window (−327 < *E*_m_ < −177 mV). Intermolecular disulfides that form within preassembled oligomers (“intracomplex” disulfides) have identical stoichiometry in oxidized and reduced states and therefore obey the same Nernst form as intramolecular disulfides, making them thermodynamically indistinguishable in our assay. Taken together, these considerations indicate that our *E*_m_ dataset likely comprises a mixture of intramolecular and intermolecular (including intracomplex) disulfides. With respect to intramolecular sites, 356 of the 366 *E*_m_-assigned proteins contain at least two cysteines (Dataset S2), and static AlphaFold models may underestimate the ability of such cysteine pairs to approach each other in alternative conformations; EF-G, whose intramolecular disulfide is experimentally supported (18) despite distant cysteines in the AlphaFold model, illustrates this limitation. We therefore regard the *E*_m_ values as most reliable for proteins in which structural data support a short S–S distance compatible with an intramolecular disulfide, whereas sites lacking such support should be interpreted more cautiously and ideally validated individually by targeted structural or mutational analysis.

Another limitation is that many potential-response curves showed incomplete oxidation at high potentials. This phenomenon is well-known but mechanistically unresolved (20) and likely arises from at least two sources. First, a fraction of proteins in lysates may be partially denatured, and proper folding is known to promote disulfide formation, whereas misfolding hinders it (55, 56)—consistent with the peptide-specific nonresponsive fraction A in our fits. Second, isobaric-tag quantification can suffer coisolation interference leading to ratio compression, particularly problematic for the first-stage label in our two-step iodoTMT strategy. Several refinements—prefractionation, interference diagnostics/corrections (e.g., y1-ion-based), computational deconvolution, MS^3^ acquisition, and FAIMS—should mitigate these effects in future datasets (57, 58). Despite these caveats, the strong agreement with purified-protein *E*_m_ (R^2^ = 0.92) and coherent network behavior argue that the conclusions are robust.

By integrating proteome-wide *E*_m_, light/dark *E* values, TrxA-catalyzed reduction assays, and structure–property analysis, we provide a quantitative energetic framework for redox regulation in *Synechocystis*. The identification of a nonequilibrium redox hierarchy—with PRK near TrxA equilibrium but F/SBPase and CP12 oxidatively biased—offers mechanistic insight into how photosynthetic metabolism is selectively wired. The dataset motivates two immediate directions: i) identifying the oxidative partners that hold specific targets above the TrxA potential in vivo, and ii) engineering disulfide switches by manipulating local geometry or partner affinity to retune operating points. More broadly, placing switches on a common energetic scale enables predictive models of redox control and opens routes to rewiring photosynthetic regulation for metabolic engineering.

## Materials and Methods

### Cyanobacteria Strain and Growth Condition.

We used the glucose-tolerant substrain of *Synechocystis* sp. PCC 6803. Cells were grown photoautotrophically in BG-11 supplemented with 20 mM HEPES–NaOH (pH 7.5) in 200 mL baffled flasks containing 50 mL medium, shaken at 100 rpm under white LEDs (30 µmol photons m^−2^ s^−1^).

### Harvest and Native Lysate Preparation.

Cultures were collected at a constant biomass input defined by OD_730_×V = 60 mL. Cells were pelleted (8,000×*g*, 5 min, room temperature), washed once with BG-11, and all subsequent steps were performed on ice or at 4 °C unless stated. Pellets were resuspended in assay buffer (25 mM HEPES–NaOH, pH 7.9; protease-inhibitor cocktail) with 0.1-mm zirconia beads (YGB01; Yasui Kikai) using a Multi-Beads Shocker MB1001CS (Yasui Kikai) at 4 °C (2700 rpm, 60 s ON/120 s OFF, 8 cycles). After centrifugation (12,000×*g*, 10 min), the supernatant was buffer-exchanged on 3-kDa centrifugal filters (Amicon Ultra, Merck) twice into 25 mM HEPES–NaOH (pH 7.9) to remove low-molecular compounds. pH 7.9 was chosen as a representative near-physiological value because the reported cytosolic pH of *Synechocystis* remains around 7.8 to 8.0 during dark/light transitions (59).

To standardize the thiol background prior to titrations, lysates (~500 µL) were briefly reduced with DTT (final ~20 mM, 1 h, dark), then transferred to Slide-A-Lyzer MINI Dialysis Units (3.5 kDa, ThermoFisher Scientific) and dialyzed against 100 mL of 25 mM HEPES–NaOH (pH 7.9) for 2 h in the dark. The buffer was replaced twice with fresh 100 mL portions of the same buffer for a further 2 h and then overnight. This treatment is expected to lower residual DTT to the low-nanomolar range, well below the reduced DTT present in the subsequent equilibration buffers (*SI Appendix,* Table S1), and samples also underwent two additional buffer exchanges during equilibration in the DTT_red_/DTT_ox_ series. Residual DTT from the prereduction step was therefore not expected to measurably affect the imposed redox potentials. Handling of redox buffers and dialysis was performed in a glovebox where indicated to minimize adventitious oxidation.

### Redox Equilibration Across Defined DTT_red_/_ox_ Potentials.

Aliquots of the native lysate (200 µg protein) were equilibrated in a series of eight DTT_red_/DTT_ox_ buffers covering −327 to −177 mV (25 °C) (*SI Appendix,* Table S1). This diluted lysate condition was chosen to maintain a well-defined DTT_red_/DTT_ox_ potential that was not measurably perturbed by reactions with protein thiols. At near-physiological protein concentrations (∼200 mg/mL) (33), the same total protein amount would correspond to ~1 µL. Under such conditions, the reduced DTT present in the most oxidizing buffer would be at the picomole scale (e.g., 0.5 µM × 1 µL = 0.5 pmol), whereas the total cysteine content in 200 µg protein is on the order of ~10 to 20 nmol (assuming ~1 Cys per 100 residues), making the imposed DTT_red_/DTT_ox_ ratio readily perturbed by protein thiols. We therefore used diluted lysates together with repeated buffer exchange so that the DTT_red_/DTT_ox_ couple reproducibly imposed the intended potential series. Each sample underwent: i) 1 h pre-equilibration in the designated redox buffer; ii) rapid buffer exchange into fresh buffer using 3-kDa filters followed by 1 h incubation; and iii) transfer into fresh buffer and a further 2 h incubation (total exposure 4 h). All incubation steps were conducted in the dark.

### Quenching and Differential IodoTMT Labeling.

Reactions were quenched by addition of trichloroacetic acid (TCA) to 10% (w/v) (1 h on ice). Protein pellets were washed with cold acetone and processed for two-step differential iodoTMT labeling using sixplex iodoTMT reagents (ThermoFisher Scientific). Distinct reporter channels were assigned to the first and second labeling steps, and the exact channel assignments for each sample in each independent experiment are listed in *SI Appendix,* Table S1. First, pellets were resuspended in labeling buffer-1 (8 M urea, 20 mM HEPES pH 8.0, 1 mM EDTA, 0.01% Triton X-100) containing iodoTMT to alkylate reduced thiols (1.5 h, 30 °C, dark). Proteins were then reprecipitated with cold acetone to remove excess reagent, resolubilized in reducing buffer (8 M urea, 20 mM HEPES pH 8.0, 1.5 mM TCEP; 30 min, 30 °C) to reduce previously oxidized thiols, and labeled again with iodoTMT in labeling buffer-2 (8 M urea, 20 mM HEPES pH 8.0, iodoTMT; 1.5 h, 30 °C, dark). This sequential alkylation–reduction–alkylation strategy is directly analogous to the differential iodoTMT scheme detailed by Nietzel et al. and was implemented here for multipotential titrations of native lysates and determination of cellular reduced/total ratios (60).

### Proteolysis, Anti-TMT Immune-Enrichment, and Desalting.

Labeled proteins were digested overnight at 37 °C with Mass Spec grade trypsin (Trypsin/Lys-C Mix, Promega) in 50 mM Tris-HCl pH 8.0. Peptides were acidified (0.5% TFA), desalted on C18 spin columns (Pierce™ C18 Spin Columns, ThermoFisher Scientific), and iodoTMT-peptides were enriched using immobilized anti-TMT resin (ThermoFisher Scientific) with overhead rotation at 4 °C (overnight). The resin was washed extensively with tris-buffered saline (TBS) and water, and peptides were eluted with the manufacturer’s TMT elution buffer, dried, reconstituted in 0.1% formic acid, and subjected to LC–MS/MS. Enrichment and elution conditions followed vendor guidance.

### LC–MS/MS Analysis.

Analyses were performed on a Vanquish Neo UHPLC coupled to an Orbitrap Exploris 240 (ThermoFisher Scientific) via a 75 µm × 15 cm C18 column (3 µm; Nikkyo Technos). Injection volume was 4 µL. Mobile phases were A: 0.1% formic acid in water and B: 0.1% formic acid in acetonitrile/water; flow, 300 nL min^−1^. The gradient was 6% B to 52% B over 105 min, ramp to 100% B by 112 min, hold to 120 min. MS1: resolution 60,000 at m/z 200, m/z 375 to 1,500, AGC target 3 × 10^6^, 100 ms max ion time, RF lens 7%. Data-dependent HCD MS2: isolation window 0.7 m/z, NCE 38, resolution 30,000, first mass m/z 120, 100 ms max ion time; dynamic exclusion 20 s.

### Estimation of *E*_m_ from Multipotential Titrations.

Reporter-ion intensities corresponding to the first and second iodoTMT labels were extracted from MS^2^ spectra. For each cysteine-containing peptide, the reduced fraction (*f*_red_) was calculated as

$$f_{\mathrm{red}}=\frac{I_{label1}}{I_{label1}+I_{label2}}.$$

Because *f_red_* was calculated from the first and second labels for the same peptide, peptide-specific differences in absolute labeling efficiency are largely normalized within each peptide. Only peptides quantified at all eight redox potentials were included for fitting. The *f*_red_(*E*) values were fitted by nonlinear least-squares regression to an extended Nernst function that incorporates a nonresponsive fraction (A) to account for the incomplete oxidation plateaus often observed at high potentials in protein redox titrations:

$$f_{\mathrm{red}}=A+\left(1-A\right)\frac{\left(\frac{{\mathrm{DTT}}_{\mathrm{red}}}{{\mathrm{DTT}}_{\mathrm{ox}}}\right)}{K_{\mathrm{eq}}+\left(\frac{{\mathrm{DTT}}_{\mathrm{red}}}{{\mathrm{DTT}}_{\mathrm{ox}}}\right)}.$$

Data were accepted when the correlation coefficient *r*^2^ > 0.9, the resulting midpoint potential satisfied −327 < *E*_m_ < −177, and A < 0.5. The equilibrium constant (K_eq_) obtained from the best-fit curve was used to calculate the *E*_m_ according to the Nernst equation:

$$E_{m}=E_{m,DTT}-\frac{\mathrm{RT}}{2F}\ln K_{\mathrm{eq}},$$

where R is gas constant, T the absolute temperature, F the Faraday constant, and *E*_m,DTT_ the standard midpoint potential of DTT (−0.327 V at pH 7.0).

### Determination of Absolute Reduced/Total Ratios in Cells Under Light and Dark.

*Synechocystis* cells were taken from cultures maintained under continuous illumination and were not entrained by prior light/dark cycling. Cells were resuspended in fresh BG-11 to final OD_730_ = 0.2 in 20 mL total volume and transferred to 50 mL tubes. For the light condition, suspensions were incubated for 2 h at 30 °C under white LEDs (50 µmol photons m^−2^ s^−1^); for the dark condition, suspensions were incubated for 2 h at 30 °C in darkness. To preserve the illumination state at quench, 100% TCA was added directly to the culture to 10% (w/v) final, and samples were placed on ice for 1 h. Afterward, samples were processed and analyzed as described above.

### Intracellular Operational Potentials *E* and Equilibration Index (EI).

For each cysteine site analyzed under light and dark conditions, the intracellular (operational) potential *E*_X_ was calculated from its site-specific midpoint potential *E*_m_,_X_ and the absolute reduced/total fraction *f_X_* determined by iodoTMT labeling using the Nernst equation for a two-electron disulfide/dithiol couple:

$$E_{X}=E_{m,X}+\frac{\mathrm{RT}}{2F}\ln(\frac{1-f_{X}}{f_{X}}).$$

The uncertainty of *E*_X_ was estimated by propagating the experimental errors of *E*_m_,_X_ and of *f_X_* using the delta method, expressed as

$${\mathrm{SE}}_{E_{X}}=\sqrt{{\mathrm{SE}}_{E_{m},X}^{2}+(\frac{\mathrm{RT}}{\mathrm{nF}}{)}^{2}{\mathrm{SE}}^{2}[ln\frac{1-f_{X}}{f_{X}}]}.$$

The same procedure was applied to obtain the potential and SE for TrxA (*E*_TrxA_ and SE_ETrxA_). To evaluate the degree of thermodynamic equilibration between each cysteine site and TrxA, the potential difference was defined as Δ*E*=*E*_X_−*E*_TrxA_, with its SE calculated as

$$SED=\sqrt{{\mathrm{SE}}_{E_{X}}^{2}+{\mathrm{SE}}_{E_{\mathrm{TrxA}}}^{2}}.$$

Degrees of freedom were approximated by the Welch–Satterthwaite equation using replicate counts for *f_X_* and *f_TrxA_*:

$$df=\frac{({\mathrm{SE}}_{E_{X}}^{2}+{\mathrm{SE}}_{E_{\mathrm{TrxA}}}^{2}{)}^{2}}{\frac{{\mathrm{SE}}_{E_{X}}^{4}}{n_{X}-1}+\frac{{\mathrm{SE}}_{E_{\mathrm{TrxA}}}^{4}}{n_{\mathrm{TrxA}}-1}},$$

where *n*_X_ is the number of biological replicates for that condition. The half-width of the 90% CI (CI) for Δ*E* was expressed as t_0.95,df_⋅SED, where t_0.95,df_ is the two-sided Student’s *t*-critical value.

The proximity of each site to thermodynamic equilibrium with TrxA was quantified by defining an equilibration Index (EI) that integrates the CI and a practical tolerance window δ as follows:

$$EI=\max\left(0,1-\frac{\max\left(0,\left|\Delta E\right|-t_{0.95,df}\cdot SED\right)}{\delta }\right).$$

We adopted δ = 5 mV to define near-equilibrium between thiol–disulfide pairs. This tolerance corresponds to the potential shift caused by a 0.1-unit pH variation (≈ 5.9 mV per 0.1 pH for 2H^+^/2e^−^ reactions) and lies within the typical reproducibility (±3 to 6 mV) of purified-protein redox titrations and iodoTMT-based measurements. Thermodynamically, 5 mV (≈ 0.97 kJ mol^−1^ = 0.4 RT at 30 °C) represents a minor driving force, thus providing a conservative yet physiologically meaningful threshold for declaring two partners in redox equilibrium.

An EI value of 1 indicates that the 90% CI of Δ*E* includes zero, meaning the site’s potential is statistically indistinguishable from TrxA (i.e., at equilibrium). EI decreases linearly as ∣Δ*E*∣ moves outside the confidence range, reaching 0 when the deviation equals the tolerance δ. Thus, EI ranges from 0 to 1 and represents how closely a site operates at the potential of TrxA after accounting for measurement uncertainty. Sites were classified as near equilibrium when EI > 0.5, corresponding to a departure from equilibrium of less than half of δ beyond the 90% CI. Unless otherwise specified, this criterion was required to hold under both light and dark conditions.

### Statistics and Reproducibility.

Unless indicated otherwise, values represent means from three biological replicates, defined as follows. For the proteome-wide *E*_m_ determination (Figs. 1 and 2), replicates correspond to three independent experiments performed on different days using independently grown *Synechocystis* cultures. For the in vivo light/dark red/total quantification (Fig. 5), replicates correspond to independent cultures grown in separate flasks and sampled on the same day under identical conditions. Error propagation for *E* and EI followed the procedures described in the EI subsection. Raw LC–MS data and search parameters are deposited in the jPOST database at https://repository.jpostdb.org/ and can be accessed with the accession number JPST004232.

### Plasmid Construction (Template and Cloning Strategy).

The coding sequences for *Synechocystis* sp. PCC 6803 *trxA* (*slr0623*), *sll1549* (N-terminal transmembrane region removed, Δ1 to 45), and *sll1835* were PCR-amplified from the *Synechocystis* genomic DNA. For CP12 (*ssl3364*) and PRK (*sll1525*), synthetic genes were designed in which the N-terminal regulatory disulfide cysteine pair was replaced with serine residues. The gene fragments were codon-optimized for *E. coli* and synthesized commercially (GenScript). Each fragment was cloned into pET-derived expression vectors carrying an N-terminal 6× His tag using the In-Fusion Snap Assembly system (Takara Bio). For constructs that failed to express adequately under standard conditions (*trxA*, *cp12,* and *prk*), an N-terminal SKIK tag was fused to enhance protein expression, as previously reported to improve the yield of difficult-to-express proteins (61). All constructs were verified by DNA sequencing before use.

### Heterologous Expression in *E. coli* and His-Tag Purification.

Expression plasmids were transformed into *E. coli* BL21(DE3). Single colonies were inoculated into LB medium with antibiotic and grown overnight. Cultures were diluted to OD_600_ = 0.05 in 20 mL LB medium with antibiotic in baffled flasks and incubated at 37 °C, 200 rpm to OD_600_ ≈ 0.5 to 1.0, then induced with 0.2 mM IPTG. Cultures were shifted to 18 °C, 200 rpm, overnight. Cell pellets were resuspended in binding buffer (20 mM Tris–HCl pH 8.0, 100 mM NaCl, 5 mM imidazole) and disrupted by bead-beating (three cycles, 30 s ON/30 s OFF) at 4 °C, followed by 14,000×*g*, 20 min, 4 °C. Clarified lysates were incubated with TALON metal affinity resin (Takara Bio) for 1 h at 4 °C with gentle rotation. Resin was washed three times with wash buffer-1 (20 mM Tris–HCl pH 8.0, 100 mM NaCl, 10 mM imidazole) and once with wash buffer-2 (same but 20 mM imidazole), and proteins were eluted with elution buffer (20 mM Tris–HCl pH 8.0, 100 mM NaCl, 500 mM imidazole). Samples were buffer-exchanged by ultrafiltration.

### Redox Titration of Purified Proteins to Determine *E*_m_.

*E*_m_ of purified proteins were determined as described previously (37). Briefly, eight redox buffers spanning −327 to −177 mV were prepared by mixing reduced and oxidized forms of DTT at defined ratios (total DTT 50 mM). The potential *E* was calculated from the DTT couple using its known *E*_m_ (−327 mV pH 7.0) and the Nernst equation at 25 °C. Purified CP12_SSCC_ and PRK_SSCC_ were equilibrated in each redox buffer for 3 h at 25 °C. Reactions were TCA-quenched (final 10% w/v), pellets washed with cold acetone, and resuspended for free-thiol labeling with AMS (4-acetamido-4′-maleimidylstilbene-2,2′-disulfonate), enabling visualization of redox state–dependent mass shifts on nonreducing SDS–PAGE. Labeled samples were resolved by nonreducing SDS–PAGE and stained with CBB. Band intensities for oxidized vs. reduced species were quantified by imageJ. Data were fit to the Nernst function for a 2-electron disulfide/thiol couple to obtain *E*_m_.

### TrxA-Dependent Electron-Transfer Assays.

CP12_SSCC_ (1 μM) or PRK_SSCC_ (0.4 μM) was incubated in 25 mM HEPES–NaOH pH 7.9 with DTT, CuCl_2_, and/or TrxA (0.4 μM). After incubation at 25 °C for 30 min, reactions were TCA-quenched (10%), and pellets were processed for AMS (for CP12_SSCC_) or MAL-dPEG®_4_-(m-dPEG®_24_)_3_ (for PRK_SSCC_; Vector Laboratories, Inc.) labeling and nonreducing SDS–PAGE exactly as in the *E*_m_ determinations.

## Supplementary Material

Appendix 01 (PDF)

Dataset S01 (XLSX)

Dataset S02 (XLSX)

Dataset S03 (XLSX)

Dataset S04 (XLSX)

## Data Availability

Proteomics data have been deposited in the jPOST repository under accession number JPST004232 (62). All study data are included in the article and/or supporting information.
