# Supplementary material for: Systematic determination of disulfide bond reduction potentials reveals a nonequilibrium redox hierarchy in cyanobacteria
Source: Proc Natl Acad Sci U S A. 2026 May 19;123(21):e2600150123. doi: 10.1073/pnas.2600150123 (PMC13214033; doi:10.1073/pnas.2600150123)
Supplement: Supplementary file 1 — Appendix 01 (PDF) [file pnas.2600150123.sapp.pdf]

Supplementary Information for:

Title: Systematic determination of disulfide bond reduction potentials reveals a non-equilibrium redox hierarchy in cyanobacteria

Authors: Kenya Tanaka[a,b,c,1], Akihiko Kondo[b], Tomohisa Hasunuma[a,b,d,e,1]

a. Engineering Biology Research Center, Kobe University, 1-1 Rokkodai, Nada, Kobe 657-8501, Japan
b. Graduate School of Science, Technology and Innovation, Kobe University, 1-1 Rokkodai, Nada, Kobe 657-8501, Japan
c. Research Center for Solar Energy Chemistry, Graduate School of Engineering Science, Osaka University, Toyonaka, Osaka 560-8531, Japan
d. RIKEN Center for Sustainable Resource Science, 1-7-22 Suehiro, Tsurumi, Yokohama, Kanagawa 230-0045, Japan
e. Department of Chemical Science and Engineering, Faculty of Engineering, Kobe University, 1-1 Rokkodai, Nada, Kobe 657-8501, Japan

[1]To whom correspondence should be addressed.
Email address: tanaka@emerald.kobe-u.ac.jp, hasunuma@port.kobe-u.ac.jp.
Telephone: 078-803-6190

This PDF file includes:
Figures S1 to S8
Legends for Datasets S1 to S4
Table S1

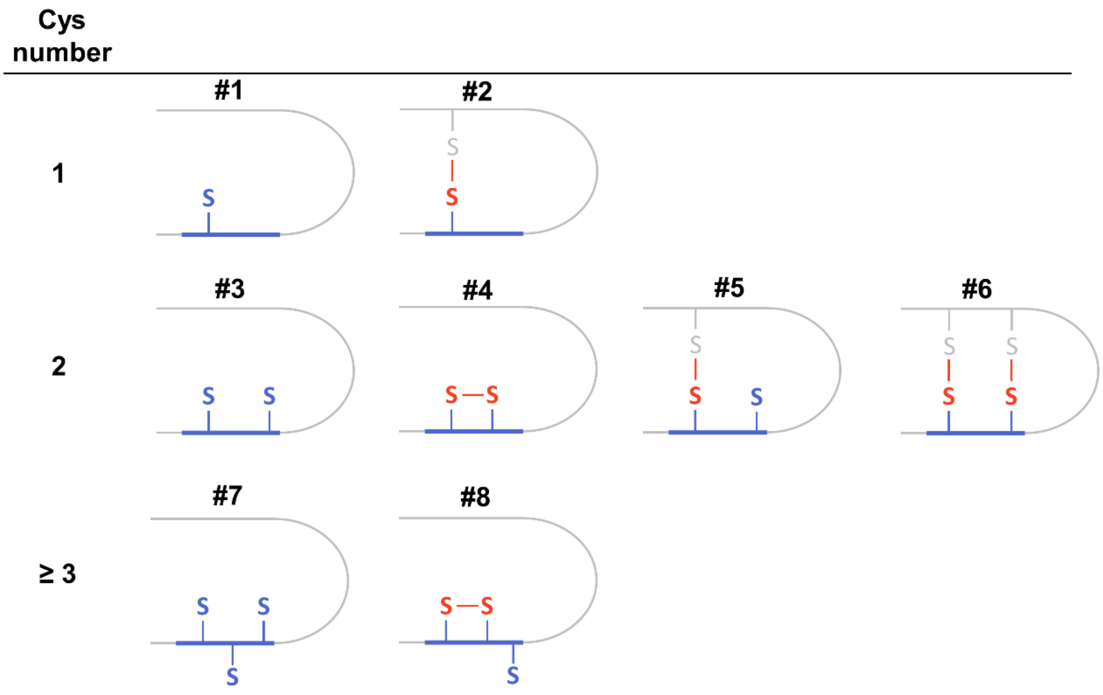

Fig. S1. Schematic classification of E_m-defined cysteine peptides by peptide cysteine content and inferred intramolecular disulfide topology. The cartoon is a schematic summary of how cysteine-containing peptides were categorized after peptide-level E_m determination and AlphaFold-based structural interpretation. The blue horizontal segment denotes the detected cysteine-containing peptide quantified by LC–MS/MS, whereas the gray line denotes the full polypeptide chain of the protein. Blue "S" indicates a reduced cysteine residue (SH or S−), and red "S" connected by a red bond indicates sulfur atoms participating in a disulfide bond in the detected cysteine-containing peptide. Gray "S" indicates sulfur atoms involved in the disulfide bond outside the detected peptide but within the same polypeptide chain.

Peptides were classified into eight categories (#1–#8) according to the number of cysteines in the detected peptide (1, 2, or ≥3) and the location of any putative intramolecular disulfide inferred from AlphaFold-based S–S distance criteria. Category #1, single-cysteine peptide with no resolvable intramolecular disulfide; #2, single-cysteine peptide whose cysteine forms a putative intramolecular disulfide with a partner outside the detected peptide; #3, two-cysteine peptide with no resolvable intramolecular disulfide; #4, two-cysteine peptide in which the two detected cysteines form a putative intramolecular disulfide within the same peptide; #5, two-cysteine peptide in which one detected cysteine forms a putative intramolecular disulfide with a partner outside the peptide; #6, two-cysteine peptide in which both detected cysteines form putative intramolecular disulfides with partners outside the peptide; #7, peptide with ≥3 cysteines and no resolvable intramolecular disulfide; and #8, peptide with ≥3 cysteines containing one putative intramolecular disulfide plus at least one additional reduced

cysteine. Thus, categories #2, #4, #5, #6, and #8 represent peptides for which at least one putative intramolecular disulfide can be assigned, whereas for categories #1, #3, and #7 the presence of an intramolecular disulfide cannot be resolved from current structural information.

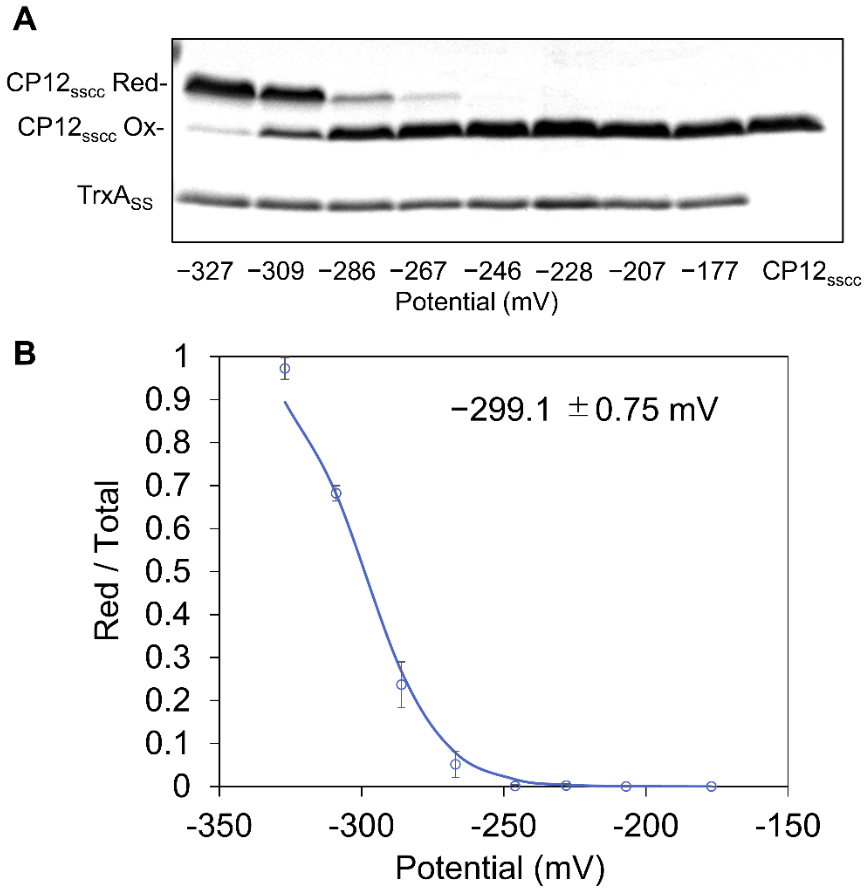

Fig. S2. Catalytically inactive TrxA does not shift the apparent E_m of CP12$_{SSCC}$. (A) Non-reducing SDS–PAGE of CP12$_{SSCC}$ equilibrated across the eight defined DTT_red/DTT_ox buffer potentials in the presence of catalytically inactive TrxA$_{SS}$, in which the active-site cysteines of TrxA were replaced with serines. Redox states were visualized after thiol labeling. (B) Nernst plot derived from gel quantification, yielding an apparent E_m of −299.1 ± 0.75 mV. Unlike wild-type TrxA, TrxA$_{SS}$ did not shift the apparent E_m of CP12$_{SSCC}$ toward the proteome-wide value, indicating that catalytic activity rather than binding alone is required for the TrxA-dependent convergence. Error bars denote SD (n = 3).

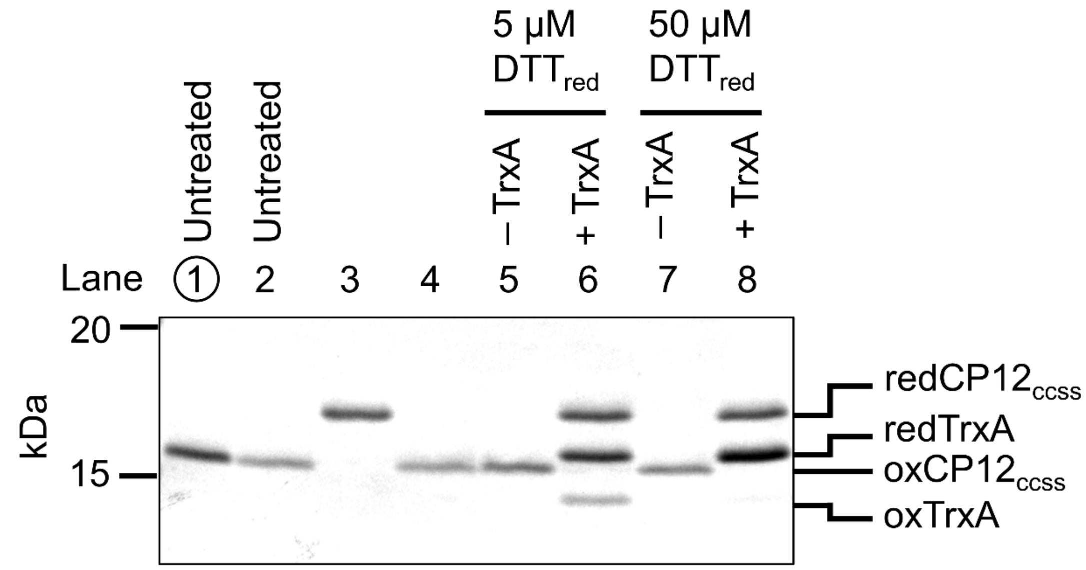

Fig. S3. The N-terminal cysteine pair of CP12 can also be reduced by TrxA. Non-reducing SDS–PAGE analysis of the $CP12_{CCSS}$ mutant, in which the C-terminal cysteine pair was replaced and the N-terminal pair was retained. Oxidized $CP12_{CCSS}$ was incubated under the same low-reductant conditions used for the C-terminal pair assay, with 5 or 50 μM reduced DTT (DTT_{red}) in the presence or absence of TrxA. The appearance of the reduced $CP12_{CCSS}$ band in the TrxA-containing reactions indicates that the N-terminal cysteine pair can also be reduced by TrxA.

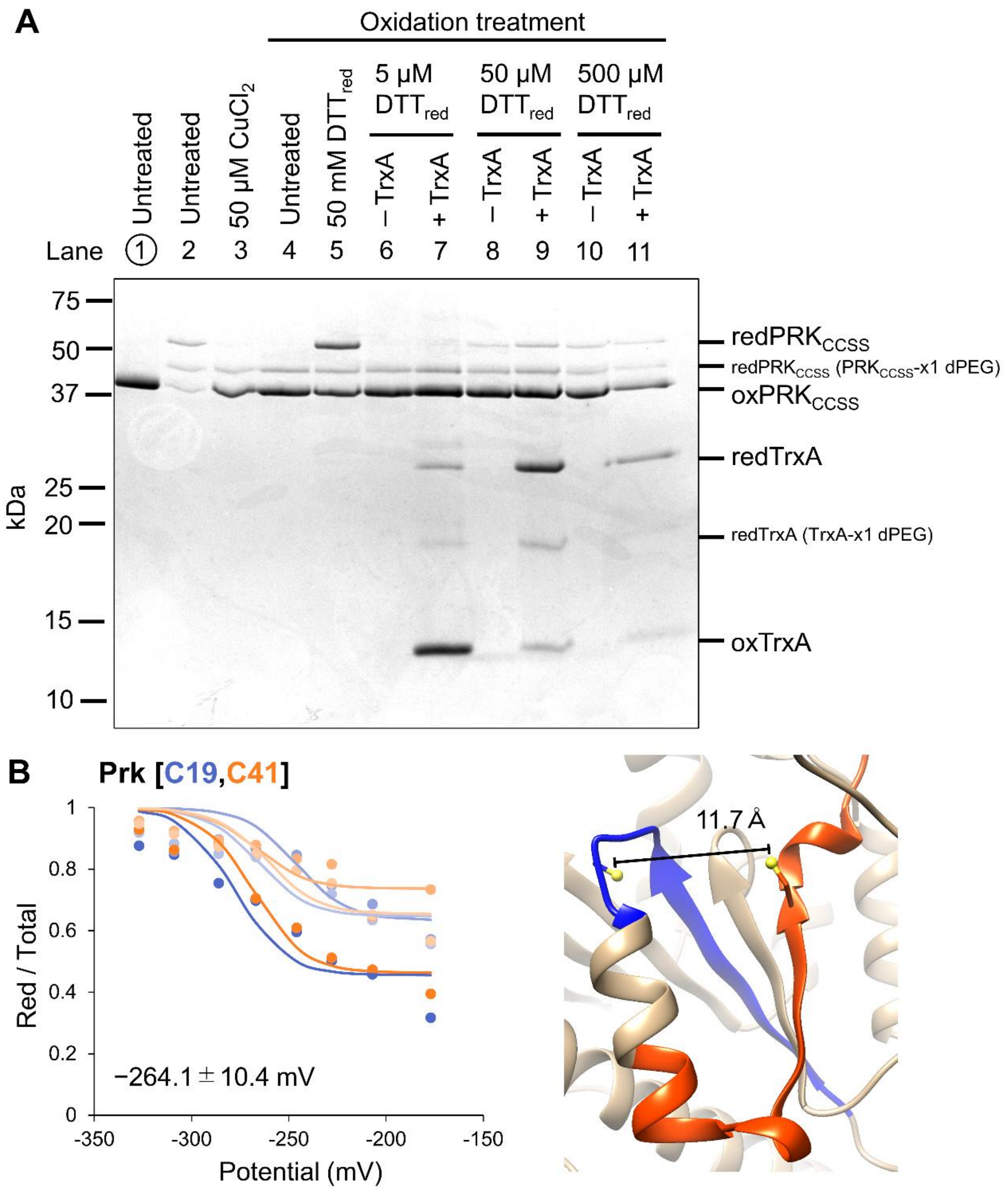

Fig. S4. The N-terminal cysteine pair of PRK is only weakly responsive to TrxA and was excluded from the main E_m dataset. (A) Non-reducing SDS–PAGE of PRK_{CCSS}, in which the C-terminal regulatory cysteine pair was replaced and the N-terminal pair was retained. After partial oxidation, PRK_{CCSS} was incubated with the indicated concentrations of DTT_red in the presence or absence of TrxA. In contrast to the C-terminal pair, the N-terminal pair showed little TrxA-dependent reduction. The singly tagged reduced PRK species is indicated. (B) Potential-dependent Red/Total plot and AlphaFold-based structural view of the N-terminal cysteine pair of PRK, yielding a fitted reference

E_m of −264.1 ± 10.4 mV. Because the fit did not satisfy the acceptance criterion for the non-responsive fraction, this pair was not included in the main E_m dataset.

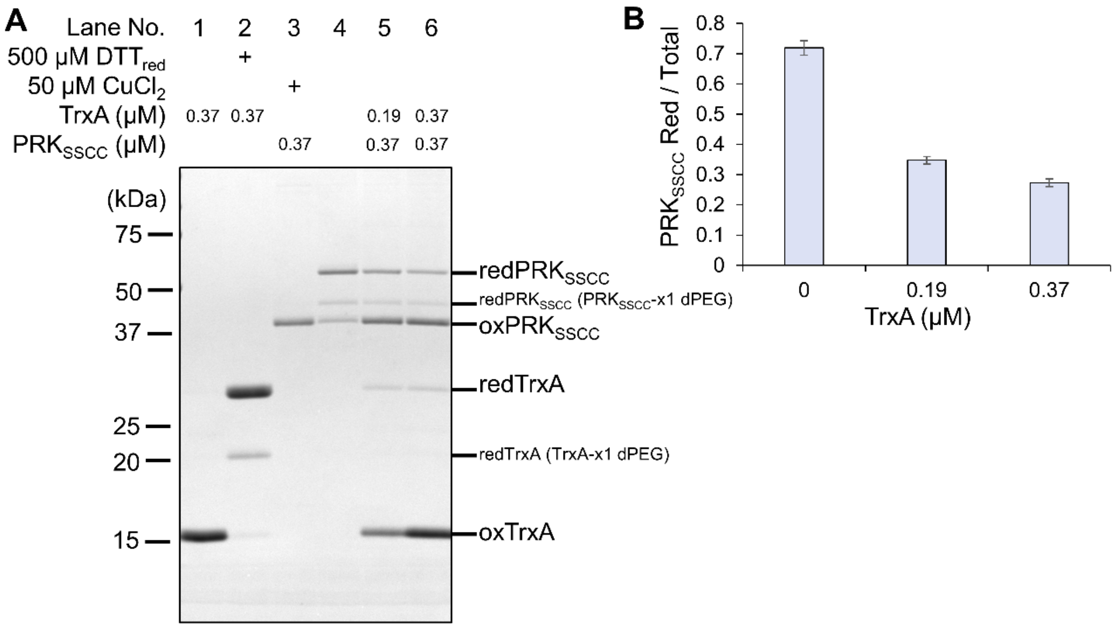

Fig. S5. Reduced PRK_{SSCC} transfers electrons to oxidized TrxA. (A) Non-reducing SDS–PAGE showing reactions containing reduced PRK_{SSCC} and oxidized TrxA at the indicated concentrations. Formation of reduced TrxA accompanied by oxidation of PRK_{SSCC} demonstrates reversible electron transfer between the two proteins. (B) Quantification of the PRK_{SSCC} reduced fraction from gel band intensities. Increasing oxidized TrxA lowered the reduced fraction of PRK_{SSCC}, supporting the interpretation that PRK can, in principle, be oxidized via TrxA. Error bars denote SD (n = 3).

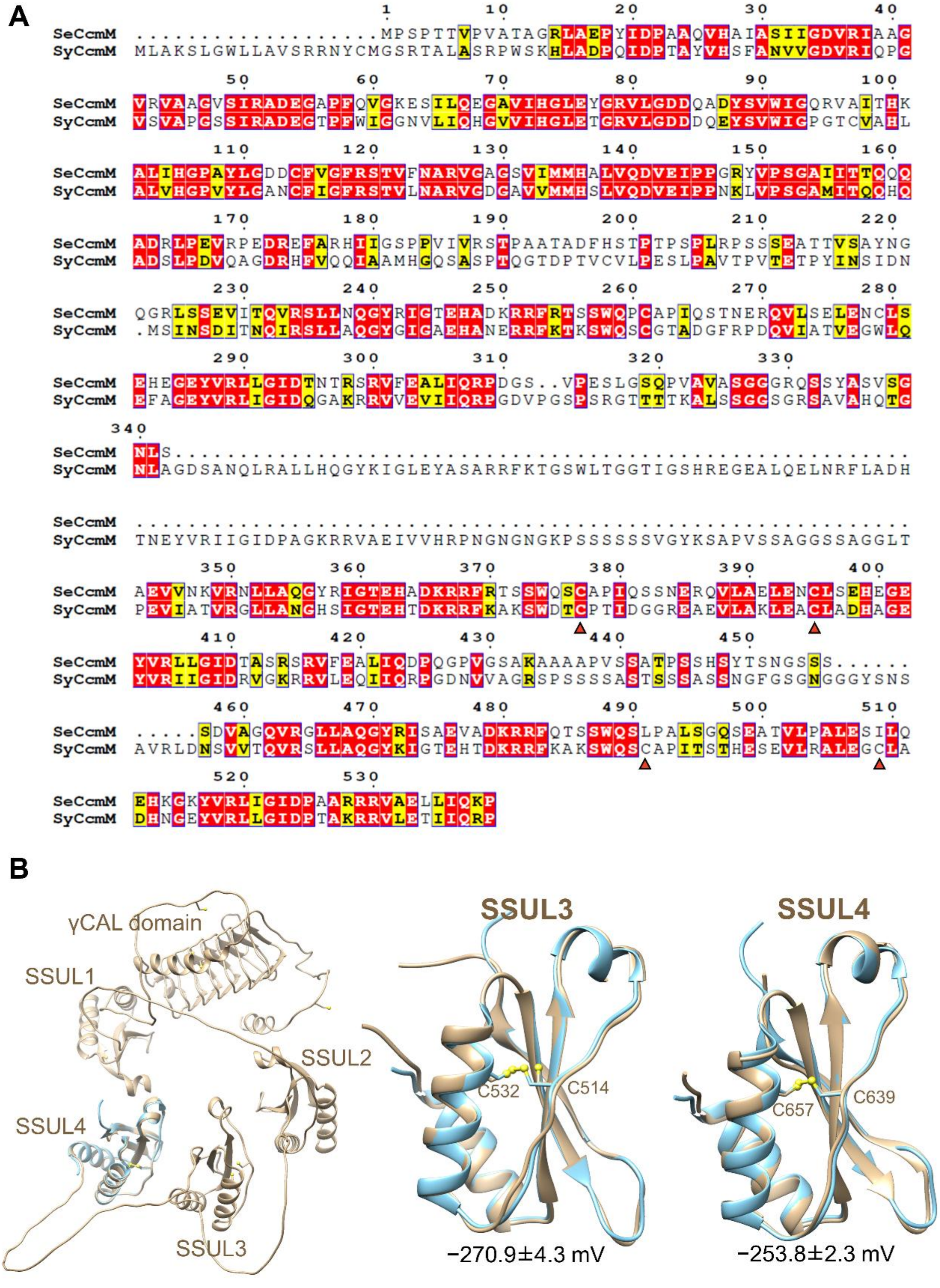

Fig. S6. Redox-relevant cysteine pairs in CcmM of *Synechocystis* sp. PCC 6803. (A) Sequence alignment of CcmM from *Synechococcus elongatus* PCC 7942 (SeCcmM) and *Synechocystis* sp. PCC 6803 (SyCcmM). Conserved residues are highlighted, and cysteine positions relevant to the detected redox-active pairs are indicated by red triangles. (B) Domain interpretation and structural comparison

of CcmM small subunit-like (SSUL) modules. The full-length AlphaFold2 model of SyCcmM is shown at left, indicating the γ-class carbonic anhydrase-like (γCAL) domain and four SSUL modules. Structural overlays at center and right compare the putative SSUL3 and SSUL4 regions of SyCcmM with the SeCcmM SSUL structure (PDB: 6HBA). In these panels, the SyCcmM AlphaFold2 model is shown in beige and the SeCcmM structure is shown in light blue. Peptides containing C514 and C532 or C639 and C657 were detected in the proteome-wide E_m dataset, yielding E_m values of −270.9 ± 4.3 mV for the putative SSUL3 pair and −253.8 ± 2.3 mV for the putative SSUL4 pair. These assignments differ from the previously characterized PCC 7942 SeCcmM disulfides, which reside in SSUL1 and SSUL2.

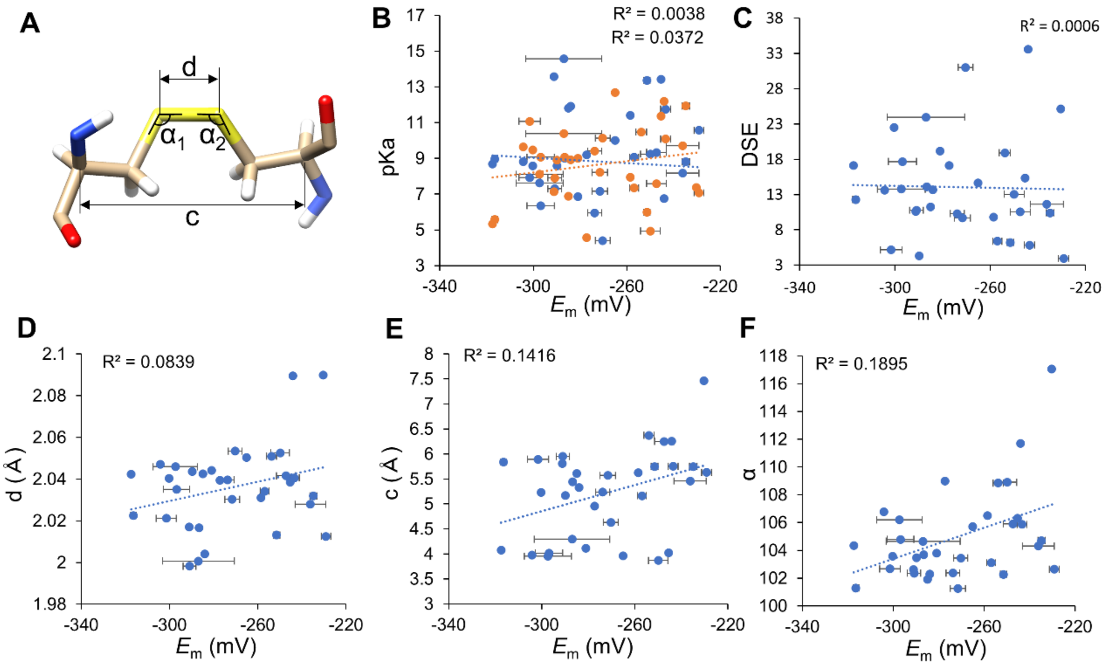

Fig. S7. Correlation of E_m with structural and physicochemical parameters of disulfide bonds.

(A) Definition of geometric descriptors for AlphaFold-predicted intramolecular disulfides: S–S distance *d*, Cα–Cα distance *c*, and dihedral angles α_1 and α_2. (B) Scatter plots of E_m versus predicted pK_a values (PROPKA) for each cysteine in the disulfide (orange and blue symbols for the two Cys residues), showing no obvious correlation (R^2 values indicated). (C) E_m versus dihedral strain energy (DSE) calculated from an empirical dihedral potential, also showing no detectable correlation. (D–F) E_m versus S–S distance *d*, E_m versus Cα–Cα distance *c* and E_m versus α angle, respectively, illustrating weak correlation with *c* and *α*. Error bars represent SD for E_m (n = 3); dashed lines indicate linear regressions.

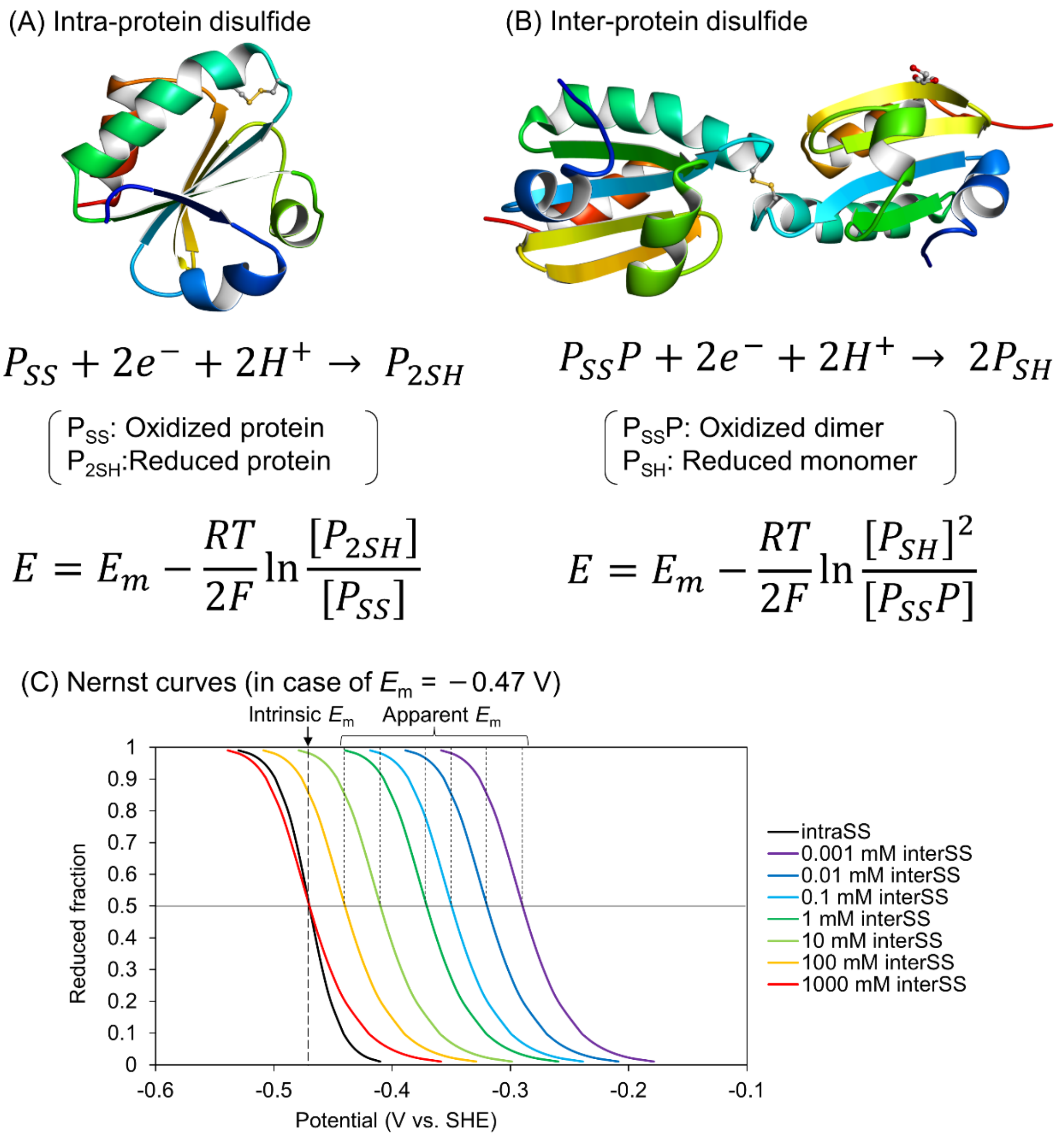

Fig. S8. Nernst behavior of intra- versus inter-protein disulfides and dependence on protein concentration.

(A) Scheme and Nernst equation for an intraprotein disulfide, where oxidized protein (P_{SS}) is reduced to the dithiol form (P_{2SH}). (B) Scheme and Nernst equation for an interprotein disulfide, where an oxidized dimer ($P_{SS}P$) is reduced to two monomeric dithiols ($2P_{SH}$). (C) Simulated Nernst curves for an intrinsic E_m =−0.47 V disulfide, comparing an intraprotein disulfide (black) with interprotein disulfides at a range of total protein concentrations (0.001–1000 mM). As concentration decreases, the apparent midpoint potential of the interprotein disulfide shifts progressively to more positive values relative to the intrinsic E_m, illustrating the strong concentration dependence of intermolecular disulfide titrations.

Legends for Datasets S1 to S4

Dataset S1. Peptide-level reduced fractions across the eight imposed DTT_{red}/DTT_{ox} potentials. Excel file providing the underlying peptide-level Red/Total values used for E_m fitting in each of the three independent experiments. Each experiment is presented in a separate column block and includes the protein name, peptide coordinates, modification state, Red/Total values measured at the eight imposed redox potentials, and the fitted parameters from the extended Nernst model, including the non-responsive fraction (A), fitted E_m, equilibrium constant, and R^2. This dataset provides a direct view of the measurements underlying Fig. 1B.

Dataset S2. Accepted E_m-defined cysteine-containing peptides from the proteome-wide titration dataset. Excel file listing all peptide-level entries that satisfied the acceptance criteria for E_m determination after fitting across the eight imposed redox potentials and removal of redundant peptide forms. For each entry, the dataset includes protein and gene annotations, peptide coordinates, cysteine positions, AlphaFold-based nearest-cysteine assignments, minimum S–S distance, mean E_m, E_m SD, and the number of independent experiments in which an E_m value was obtained (N). Type_3A and Type_4A indicate peptide-class assignments based on structural interpretation using S–S distance criteria, as summarized in Fig. S1. The "SS pair" column links paired entries assigned to the same inferred intramolecular disulfide when the two cysteines were detected on separate peptides.

Dataset S3. Light–dark absolute red/total ratios and statistics for cysteine-containing peptides. Excel file reporting absolute reduced/total (red/total) fractions for cysteine-containing peptides quantified *in vivo* under light and dark conditions. Columns include: UniProt ID, peptide position (Peptide detected), protein name and gene, cysteine positions in the sequence (Cys1–Cys4), mean red/total under light and dark (Av_Light, Av_Dark), corresponding standard deviations (Std_Light, Std_Dark), and t-test P values (t.test) for the light–dark difference. "Dif" indicates the difference in mean reduced fraction between light and dark (Av_Light − Av_Dark). "Dif × ($-\log_{10}$P)" is the product of this difference and the negative base-10 logarithm of the P value, and was used as a ranking metric to prioritize entries that showed both a large light–dark shift and strong statistical significance. "Rank" indicates the order of entries based on this metric. This dataset underlies the identification of light-responsive cysteine sites and the proteins highlighted in Fig. 5A,B.

Dataset S4. Site-resolved *in vivo* red/total ratios, and operational potentials.

Excel file providing the full per-site dataset used for calculation of intracellular operational potentials (E) under light and dark. Columns include: UniProt ID, peptide position (Peptide detected), protein name, individual E_m estimates (Em_1–Em_6), red/total ratios for up to three biological replicates in light (L1–L3) and dark (D1–D3), and derived quantities: light and dark operational potentials (E_L,

E_D), their standard deviations (SD_L, SD_D), two-sided P values for the difference from the E_m value (two-sided p_L, two-sided p_D), and Equilibration Index values in light and dark (EI_L, EI_D). This dataset is used to evaluate equilibrium vs. non-equilibrium organization of sites with respect to TrxA, as shown in Fig. 5C,D and related analyses.

Table S1. Composition of the eight DTT_{red}/DTT_{ox} equilibration buffers and iodoTMT channel assignments.

Sample No.		1	2	3	4	5	6	7	8
Potential (mV)		-327	-309	-286	-267	-246	-228	-207	-177
Ox DTT (mM)		25	40	48	49.5	49.9	49.975	49.995	49.9995
Red DTT (mM)		25	10	2	0.5	0.1	0.025	0.005	0.0005
100 mM Ox DTT (μL)		100	160	192	198	199.6	199.9	199.98	200
100 mM Red DTT (μL)		100	40						
10 mM Red DTT (μL)				80	20				
1 mM Red DTT (μL)						40	10		
0.1 mM Red DTT (μL)								20	2
PROTEIN (μg)		200	200	200	200	200	200	200	200
TOTAL (μL)		400	400	400	400	400	400	400	400
N1	1st label	127	128	126	127	128	126	127	127
	2nd label	130	131	129	130	131	129	130	130
N2	1st label	126	128	128	127	128	126	127	128
	2nd label	129	131	131	130	131	129	130	131
N3	1st label	126	127	128	126	127	128	126	126
	2nd label	129	130	131	129	130	131	129	129

Redox buffer series used for proteome-wide E_m determination. For each imposed potential, the table lists the target buffer potential, final concentrations of oxidized and reduced DTT, preparation volumes from stock solutions, total protein input, and final reaction volume. Reporter-channel assignments for the first and second iodoTMT labeling steps in the three independent experiments (N1–N3) are also shown.
